# Evolution of *AANAT*: expansion of the gene family in the cephalochordate amphioxus

**DOI:** 10.1186/1471-2148-10-154

**Published:** 2010-05-25

**Authors:** Jiri Pavlicek, Sandrine Sauzet, Laurence Besseau, Steven L Coon, Joan L Weller, Gilles Boeuf, Pascaline Gaildrat, Marina V Omelchenko, Eugene V Koonin, Jack Falcón, David C Klein

**Affiliations:** 1 Section on Neuroendocrinology, Program in Developmental Endocrinology and Genetics, The Eunice Kennedy Shriver National Institute of Child Health and Human Development, National Institutes of Health, Bethesda, 20892, MD, USA; 2 Laboratoire Arago, UPMC-Paris 6 and CNRS, FRE 3247 & GDR 2821, Facteurs du Milieu & Mécanismes Adaptatifs, Banyuls/Mer, 66651, France; 3 Museum National d'Histoire Naturelle (MNHN), 43 rue Cuvier, Paris, 75005, France; 4 National Center for Biotechnology Information, National Library of Medicine, National Institutes of Health, Bethesda, 20894, MD, USA

## Abstract

**Background:**

The arylalkylamine *N*-acetyltransferase (AANAT) family is divided into structurally distinct vertebrate and non-vertebrate groups. Expression of vertebrate AANATs is limited primarily to the pineal gland and retina, where it plays a role in controlling the circadian rhythm in melatonin synthesis. Based on the role melatonin plays in biological timing, AANAT has been given the moniker "the Timezyme". Non-vertebrate AANATs, which occur in fungi and protists, are thought to play a role in detoxification and are not known to be associated with a specific tissue.

**Results:**

We have found that the amphioxus genome contains seven *AANAT*s, all having non-vertebrate type features. This and the absence of *AANATs *from the genomes of Hemichordates and Urochordates support the view that a major transition in the evolution of the *AANATs *may have occurred at the onset of vertebrate evolution. Analysis of the expression pattern of the two most structurally divergent *AANAT*s in *Branchiostoma lanceolatum *(*bl*) revealed that they are expressed early in development and also in the adult at low levels throughout the body, possibly associated with the neural tube. Expression is clearly not exclusively associated with the proposed analogs of the pineal gland and retina. blAANAT activity is influenced by environmental lighting, but light/dark differences do not persist under constant light or constant dark conditions, indicating they are not circadian in nature. bfAANATα and bfAANATδ' have unusually alkaline (> 9.0) optimal pH, more than two pH units higher than that of vertebrate AANATs.

**Conclusions:**

The substrate selectivity profiles of bfAANATα and δ' are relatively broad, including alkylamines, arylalkylamines and diamines, in contrast to vertebrate forms, which selectively acetylate serotonin and other arylalkylamines. Based on these features, it appears that amphioxus AANATs could play several roles, including detoxification and biogenic amine inactivation. The presence of seven AANATs in amphioxus genome supports the view that arylalkylamine and polyamine acetylation is important to the biology of this organism and that these genes evolved in response to specific pressures related to requirements for amine acetylation.

## Background

The AANAT family is part of the large and diverse superfamily of GCN5-like acetyltransferases which use AcCoA as the acetyl donor and share a common AcCoA binding fold [1]. Members of the AANAT family share limited sequence identity and are divided into two groups: vertebrate AANATs; and, the non-vertebrate AANATs. The latter are found in fungi, protists, and bacteria and lack defining physical characteristics of vertebrate AANAT [1-3]. The biological role of vertebrate AANAT is to acetylate serotonin in the synthesis of melatonin (tryptophan → hydroxytryptophan → serotonin → *N*-acetylserotonin → melatonin) [4,5]. Vertebrate AANAT is associated with biological timing: daily changes in the activity of this enzyme regulate the daily rhythm in melatonin synthesis, which is essential for optimal temporal coordination of biological functions with night/day and seasonal changes and for photic entrainment [6]. The central role of vertebrate AANAT in biological timing has earned it the moniker 'the Timezyme' [7]. Non-vertebrate AANATs are thought to play a detoxifying role by neutralizing arylalkylamines [8] and a role in DNA biology by acetylating polyamines [9].

Genomes of vertebrates contain a single copy of the *AANAT *gene, except for teleost fish, some of which possess up to three paralogs [2], and cows which possess two paralogs (unpublished; NCBI, NIH, http://www.ncbi.nlm.nih.gov). Members of the *AANAT *family are not in the available genomes of Hemichordates and Urochordates, which leaves open the question of when vertebrate *AANAT *first appeared in chordates. A striking characteristic of vertebrate *AANAT *is that it is consistently expressed at significant levels in only two tissues, both of which are photosensitive organs, the pineal gland and retina. This fits with the evidence that pinealocytes and retinal photoreceptors evolved from a common ancestral photodetector [10-13]. The vertebrate AANAT has a neutral pH optimum and exhibits high selectivity for arylalkylamines [7].

Vertebrate *AANATs *encode proteins that have several highly conserved structural characteristics [2,8], which facilitate arylalkylamine acetylation and regulation. These features include flanking regulatory regions, which mediate rapid changes in enzyme activity; a pair of histidines which facilitate catalysis [14]; and, a proline-containing tripeptide in a floppy loop, which confers a high catalytic rate through an effect on substrate binding [15]. Vertebrate AANATs also have high selectivity for arylalkylamines conferred by the binding pocket.

Non-vertebrate type *AANAT*s are found in the genomes of most fungi, many unicellular eukaryotes, and a variety of bacteria [16]. The proteins encoded by these genes do not contain the characteristic structural features of vertebrate AANATs. Genes similar to non-vertebrate *AANAT*s are not found in vertebrates.

Amphioxus *AANATs *are of interest in understanding the evolution of vertebrate *AANAT *because cephalochordates and vertebrates form a clade [17]. Accordingly, the study of the *AANAT *from this organism might provide new insights into the transition from the non-vertebrate members of the *AANAT *family to the vertebrate forms. Sequence in the trace archives has indicated that a non-vertebrate type member of the *AANAT *family is present in amphioxus [2]. The subsequent availability of the near complete amphioxus genome [18] has allowed us to comprehensively analyze the *AANAT *family in amphioxus, as presented here. The findings of this study indicate that rapid expansion of non-vertebrate forms occurred in amphioxus and that a vertebrate form is absent. The substrate preference profile of the encoded enzymes suggests to us that they are likely to play a role in detoxification or in biogenic amine inactivation, or both.

## Methods

### Animals

*Branchiostoma floridae (B. floridae, bf) *and *Branchiostoma lanceolatum *(*B. lanceolatum, bl*) were used; the former were obtained from Gulf Specimen Marine Lab (Panacea, FL, USA) and the latter from the Bay of Banyuls sur Mer (France) [17]. *B. floridae *heads were used to generate the cDNA used to clone full length *bfAANATα *and *bfAANATδ' *that were used for enzyme characterization. *B. lanceolatum *were used for *in vivo *studies of gene expression and enzyme activity. They were cultured in sand-filtered seawater under a natural lighting cycle and temperature, as indicated elsewhere [17]. Intensity of the light was 136 lux. For the experiment with constant conditions, animals were housed for 14 days in constant light and/or darkness and then were used for the activity assay.

### Identification of amphioxus *AANAT*s

*bfAANAT *homologs were identified through a tBLASTn search of genomic sequences available at: http://genome.jgi-psf.org[18]. Seven genes for AANAT homologs (α - η were identified in genome assembly v2.0. A second allele for five of the genes was found in v1.0. Based on these sequences, primers were designed (Additional file 1) for cloning of *blAANAT *cDNAs. qPCR primers for specific gene products were based on the *B. lanceolatum *clones.

### Sequence similarity search and phylogenetic analysis

The *AANAT *homologs were identified by searching the non-redundant database of protein sequences (NCBI, NIH, http://www.ncbi.nlm.nih.gov) using the PSI BLAST program [19] and by searching the genomes and proteomes available at JGI http://genome.jgi-psf.org/euk_cur1.html using the tBLASTn and BLASTp programs, respectively. Additionally, *AANAT *homologs were detected in the genomes of the red algae *Gracilaria changii*, segmented worm *Alvinella pompejana*, Mediterranean mussel *Mytilus galloprovincialis *(NCBI, EST database) and the basal fungus *Rhizopus oryzae *http://www.broadinstitute.org/annotation/genome/rhizopus_oryzae/MultiHome.html.

Representatives from different groups including bacteria, fungi, protists, and animals were selected for phylogenetic analysis, and a multiple alignment of the respective protein sequences was constructed using the MUSCLE program [20].

A maximum likelihood phylogenetic tree was constructed using the TreeFinder program [21] by optimizing a default starting tree constructed using the Neighbor-joining method with the Whelan and Goldman (WAG) empirical model of substitutions [22] To select the best substitution model, we compared the likelihoods of trees constructed using 8 models: WAG (21), JTT [23], VT [24], BLOSUM [25], Dayhoff [26], cpREV [27], rtREV [28], PMB [29] by running the TreeFinder program [21]; this test showed the best fit with the data for the WAG model. The reliability of the internal tree branches was estimated using the LR-ELW bootstrap method [30]. A constrained large scale tree topology was created using the TreeView program [31]. Alternative tree topologies were compared with the Approximately Unbiased test [32] using the TreeFinder program.

Evolutionary distances were estimated using the distance matrix created by the PROTDIST program of PHYLIP package [33] based on a truncated alignment of AANATs and using the Jones-Taylor-Thornton (JTT) substitution model [23]. The mean differences (p-distance) and identities between the major taxonomic groups of AANATs were calculated using the MEGA program [34].

### Prokaryotic expression of recombinant proteins

pGEX 4T-1 (GE Healthcare, Piscataway, NJ) was used for the recombinant expression of full-length bfAANATα and bfAANATδ'. The constructed plasmid was sequenced to confirm identity. The construct, which produces a GST fusion protein, was transformed into *E. coli *strain BL21Gold (DE3) pLysS (Novagen, Madison, WI). The cells were grown at 37°C; when the OD_600 _= 0.6, the cultures were cooled to 25°C and isopropyl β-D-1-thiogalactopyranoside was added (final concentration = 0.2 mM). The cells were harvested by centrifugation after 12 hours of culturing (5000 × g, 30 min, 4°C) and resuspended in 2× PBS, pH 7.5, containing 10 mM DTT (Buffer A) and a mixture of protease inhibitors (Complete^®^, Roche, Indianapolis, IN). The cells were then lysed by sonication and the resulting lysate was centrifuged (8500 × g, 25 min, 4°C). The supernatant was mixed with Glutathione-Sepharose 4B affinity matrix (GE Healthcare) pre-equilibrated with Buffer A. The suspension was agitated for 1 h and then packed into a glass column. The column was washed with 5 column volumes of Buffer A, followed by 5 column volumes of buffer containing 50 mM Tris-HCl, 0.1 M sodium citrate, 5 mM DTT and 10% glycerol, pH 7.8 (Buffer B). GST fusion protein was then eluted with 5 column volumes of Buffer B containing 10 mM glutathione. Protein was concentrated and dialyzed against buffer containing 0.1 M ammonium acetate, 25 mM NaCl and 1 mM TCEP; the resulting preparation was stored at -80°C. Where indicated, purified GST-free bfAANATα was used; otherwise, the GST-bfAANATα or GST-AANATδ' fusion product was used, consistent with previous studies [15,35,36].

### SDS-PAGE

Proteins were resolved on preformed 14% Tris-glycine (1 mm) gels, using the manufacturer's protocol (NOVEX, Invitrogen) [37]. The molecular mass of the proteins was estimated using Rainbow™ markers (GE Healthcare).

### Fluorescence-based protein studies

Fluorescence measurements were done on an ISS PC1 photon-counting spectrofluorimeter (ISS, Inc., Champaign, IL) at 22°C. To study binding properties of bfAANATα protein, the interaction between bfAANATα and CoA-S-N-acetyl-7-hydroxynaphthylethylamine (CoA-HNE), a fluorescent bisubstrate inhibitor of vertebrate AANAT, was analyzed [36]. This was done by measuring CoA-HNE (1 μM)-dependent quenching of tryptophan fluorescence in 0.1 M ammonium acetate, 25 mM NaCl and 1 mM TCEP, pH 6.8 (excitation = 290 nm; emission = 310 nm). Off-rates of CoA-HNE were monitored as described [15] using protein preparations that were 90% saturated with CoA-HNE. Following a control incubation period, the probe was displaced with a 100-fold excess of another bisubstrate inhibitor, CoA-S-N-acetyltryptamine (CoA-T), that is optically inactive in this analysis [36]. The limiting value of k_off _was calculated by non-linear fitting (SigmaPlot Version 10.0, Systat Software, Inc., Point Richmond, CA) using the equation:

where A(t) is the observed fluorescence anisotropy at time 't' after the initial addition of CoA-T and k_off _is the rate constant.

### Determination of *N*-acetyltransferase activity

Recombinant bfAANATα activity was measured radiochemically by measuring [^3^H]acetyl product formed from [^3^H]acetyl-coenzyme A ([^3^H]AcCoA; GE Healthcare, UK) and an amine substrate as described [15]; or, colorimetrically by measuring CoASH formed by incubation with AcCoA and an amine substrate. CoASH is detected as the colored product formed by reaction with 5,5'-dithio-bis(2-nitrobenzoic acid) (DTNB) [35]. In the colorimetric assay, standard reactions were performed in a total volume of 100 μL containing: the substrate at indicated concentrations, 0.5 mM AcCoA, 0.05 mg/ml bovine serum albumin (BSA), and 2 mM ethylene diamine tetraacetic acid (EDTA). For analysis of GST-bfAANATα, 0.25 μg of protein was included in 0.1 M Tris buffer pH 9.5; the activity of ovine GST-AANAT, which was used as a reference standard [15], was determined by incubating 10 ng of protein in 0.1 M phosphate buffer, pH 6.8. Incubation conditions were either 80 min at 37°C for amphioxus or 30 min at 37°C for ovine AANAT. Enzymes were then inactivated by the addition of 150 μl stop solution (1 mM DTNB, 10 mM EDTA, and 3 M guanidine hydrochloride); absorbance (405 nm) was measured after 5 min incubation at room temperature. The V_max _and K_m _were calculated by non-linear fitting (Prism5 from GraphPad) using the equation:

where V is the observed enzyme velocity (activity), V_max _is the maximum enzyme velocity, S is the substrate concentration, and K_m _is the Michaelis-Menten constant.

For the measurement of enzyme activity in amphioxus head tissue, the colorimetric assay (see above) was used with 10 mM β-phenylethylamine (PEA) as a substrate and 10 μg of protein from tissue homogenized in assay buffer. For each measurement a corresponding assay was performed in the presence of 1 mM CoA-T [15], and another in the absence of PEA. The controls used included an assay without tissue homogenate and another with a heat-inactivated (65°C, 5 min) homogenate. Samples were collected at noon and midnight in the experiments studying the light/dark variations, and at noon in all other experiments.

### Quantitative measurement of *B. lanceolatum *AANAT transcripts (qPCR)

Total RNA was extracted from head and from body using a Trizol extraction kit (Invitrogen, Cergy Pontoise, France). 1 μg was incubated with 1 unit of DNase I (Roche; Meylan, France) for 20 min at 37°C. Following DNase inactivation (65°C, 10 min), the sequence was reverse-transcribed using Powerscript Reverse Transcriptase (Clontech, Mountain View, CA). qPCR of the resulting cDNAs was performed with the primer sets qAANAT (Additional file 1) using the 'Light Cycler Fast Start DNA Master SYBR Green I kit' (Roche Molecular Biochemicals, USA) in a total volume of 20 μl as follows: 95°C (10 min), followed by 35 cycles of denaturation at 95°C (10 sec), annealing at 60°C (15 sec) and extension at 72°C (15 sec). qPCR was done using a Light Cycler 1.5 (Roche Diagnostics, USA). Amplification efficiency was measured using serial dilutions of plasmids containing a fragment of the corresponding blAANAT gene. Data were normalized to the average value obtained with three housekeeping genes from *B. lanceolatum*: L17 (AY130354), L18 (AY130454) and actin (Y13663). The PCR conditions for the housekeeping genes were as indicated above except that the annealing temperature was 66°C for actin. Primers for the housekeeping genes are given in Additional file 1.

### Tissue distribution of amphioxus AANATα studied by RT-PCR

The tissue distribution of amphioxus AANATα was determined by RT-PCR. Total RNA from different parts of *B. lanceolatum *was extracted using the Trizol method (Invitrogen); 1 μg was incubated with 1 unit of DNAse I (Roche) for 20 min at 37°C. Following DNAse inactivation (65°C, 10 min), the RNA was reverse-transcribed using Powerscript Reverse Transcriptase (Clontech) PCR amplifications of the resulting cDNAs using Advantage cDNA Polymerase (Clontech) were performed with the primers for *blAANATα *(Additional file 1), and the following conditions: 95°C (1 min) followed by 30 cycles of denaturation at 94°C (20 sec), annealing at 66°C (1 min) and extension at 68°C (30 sec). In the controls, the template cDNA was replaced by either water or RNA that was not reverse-transcribed. The PCR products were loaded in a 1% agarose gel, in the presence of DNA/Hinf I marker (Promega, Madison, WI). Fragments of the expected size were extracted, subcloned in pGEM-T Easy (Promega) and sequenced.

### Tissue distribution of amphioxus AANATα studied by *in toto *hybridization

*B. lanceolatum *were collected at Argelès-sur-Mer (France) in July 2009; gametes were obtained by heat stimulation [17,38]. Eggs and embryos were fixed in 4% paraformaldehyde (PFA) in MOPS-EGTA buffer and processed for *in toto *hybridization as detailed elsewhere [39], except that the chromogenic reaction was performed using BM Purple (Roche) [40]. Two digoxigenin labeled probes corresponding to the α or δζ subtypes were designed. The former was obtained using the cloning primers cAANATαF2 and cAANATαR2 (Additional file 1) and yielded a probe of 453 bp. The latter was obtained using the cloning primers cAANATδζF2 and cAANATδζR2 (Additional file 1) and yielded a probe of 308 bp. Hybridization treatments with the different sense and antisense probes were performed simultaneously.

## Results

### Sequence analysis, phyletic distribution, and phylogeny of the AANATs

**Figure 1:**
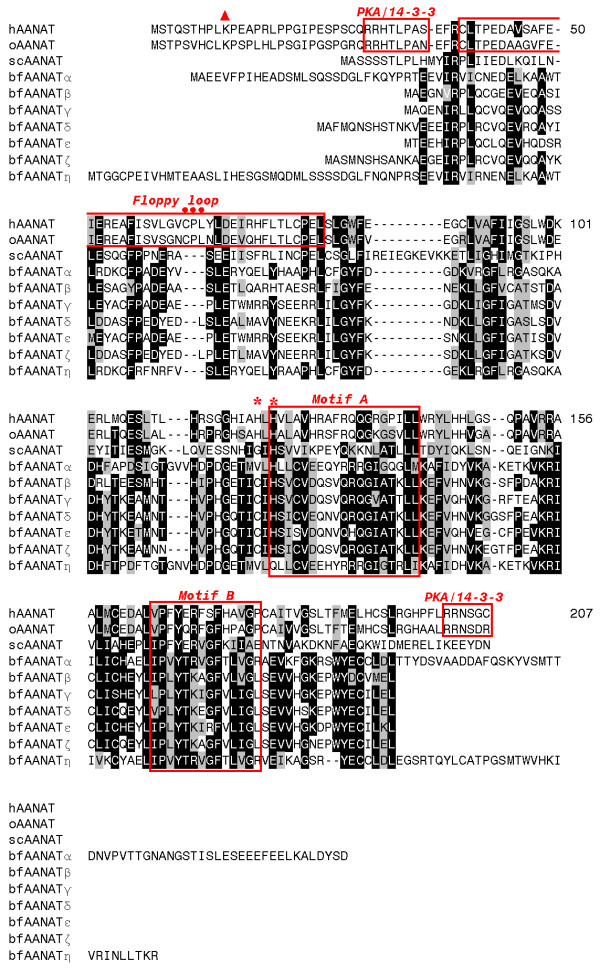
**Multiple alignment of sequences**. Alignment of AANAT sequences from human (h), ovine (o) and yeast (sc), compared with the newly identified bfAANATs. Sequences have been aligned using CLUSTALW [51], and then manually adjusted. Residues inversely highlighted in black are identical; residues highlighted in grey are similar among compared sequences. Motifs conserved in vertebrate AANATs and discussed in the text are identified with labeled red boxes. Individual residues discussed in the text are marked with red symbols. Red triangle: conserved lysine; red dots: three residues lacking in all non-vertebrate AANAT homologs; red asterisks: paired histidines involved in catalysis. See Additional file 2 for amphioxus sequence coordinates and Additional file 7 for alignment of AANAT sequences from more species.

Seven intronless *AANAT *genes were identified in the *B. floridae *genome (Figure 1). A second allele was found for 5 of the 7 genes in version v1.0 of the *B. floridae *genome assembly. The amino acid sequence identity among the amphioxus AANATs ranged from 30 to 86% excluding allele/allele comparisons (Additional file 2). The structural features which characterize vertebrate AANATs, including the flanking regulatory regions, a pair of neighboring histidines in the active site, and a tripeptide in the floppy loop, do not appear in the proteins encoded by the amphioxus *AANAT*s. The motifs A and B, which characterize members of the GCN5-like superfamily of acetyltransferases are conserved in both amphioxus and vertebrate AANATs (Figure 1).

The relative locations of the *bfAANAT*s on 3 assembly scaffolds are shown schematically in Additional file 3. The 7 genes comprise 4 groups based on sequence similarity - group 1: αη group 2: β group 3: γε group 4: δζ. Primers were prepared to amplify members of each of the four groups in *B. lanceolatum; *low sequence conservation prevented amplification of all seven. The resulting PCR products were sequenced and designated *blAANATα*, *blAANATα' blAANATγ*, and *blAANATγδ *(GenBank accession numbers FJ668653, FJ668656, FJ668654, FJ668655, respectively; α' is used to identify the α allele), according to the homology with individual *bfAANAT *genes; representatives of the other genes were not obtained in this screen. The clone *blAANATδζ *was designated this way because it could not be reliably ascribed to either the δ or ζ gene based on similarity to the *B. floridae *counterparts. The amino acid sequence identity between the cloned PCR products for the *B. lanceolatum *α, α', γ and δζ isoforms and the corresponding *B. floridae *genes was 94%, 86%, 76%, and 73%, respectively. Comparisons of the amino acid sequences of the cloned blAANATα and blAANATδζ fragments to their *B. floridae *counterparts are shown in Additional file 4.

Examination of the phyletic distribution of the detected AANAT family members shows that the family is represented not only in chordates and cephalochordates but also in some invertebrates such as the primitive metazoan *Trichoplax *(four paralogs), the segmented worms *Capitella *and *Alvinella*, and the mollusk *Mytilus*. Among non-metazoan eukaryotes, AANAT homologs were detected in different fungal lineages including basidiomycetes, ascomycetes, and the basal fungi *Rhizopus oryzae *and *Phycomyces blakesleeanus*, and in many unicellular eukaryotes (protists). The protists in which AANAT homologs were detected include representatives from three supergroups of eukaryotes, namely, plantae, chromalveolates and excavates [41]. In plantae, AANAT homologs were found in green and red algae; among chromalveolates, members of the AANAT family were present in diatoms, oomycetes, and a haptophyte *Emiliania huxleyi; *and, among excavates, an AANAT homolog was identified in the heterolobosean *Naegleria gruberi*. AANAT homologs are also present in a number of bacterial lineages including firmicutes, actinobacteria, proteobacteria and the bacteroidetes/chlorobi group (see Additional files 5 and 6).

**Figure 2:**
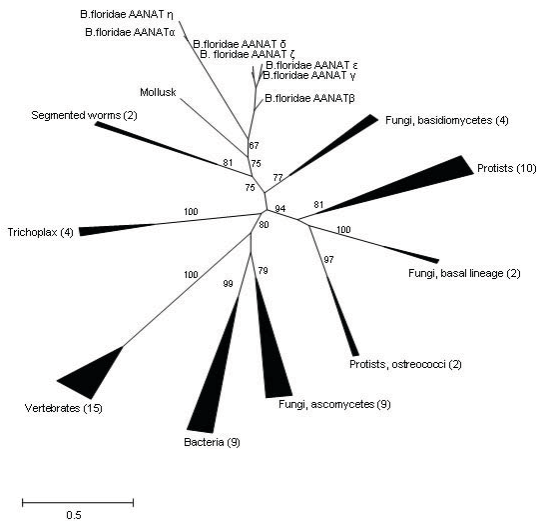
**Unrooted maximum likelihood phylogenetic tree of the AANAT family**. The scale bar shows the number of substitutions per position; the numbers in parenthesis show the number of species in the respective branches; and, the number at internal branches show the bootstrap support (%). For the list of species, the sequences in FASTA format and the multiple alignment used for the tree construction, see Additional files 5, 6 and 7.

AANATs are relatively short proteins which complicates reliable phylogenetic analysis. Phylogenetic trees were constructed based on an alignment of 65 amino acid sequences (see Additional files 7, 8, 9 and 10). Eight TreeFinder runs were performed for 8 substitution models (see Methods). The ML tree constructed with the WAG matrix was associated with the highest likelihood value. The topology of this tree is incompatible with the species phylogeny (Figure 2). The monophyly of all 7 amphioxus AANATs was moderately supported and these sequences formed an apparent clade with mollusk and segmented worm that was even more strongly supported (Figure 2). However, beyond this invertebrate branch, there were many anomalies in the tree. In particular, vertebrates clustered with ascomycetes and bacteria, whereas the position of *Trichoplax *remained uncertain. The monophyly of fungi was not recovered, and ostreococii were separated from another green alga represented in the selected set of protists (Figure 2).

We tested the statistical compatibility of the AANAT tree with the large-scale organismal phylogeny of eukaryotes using the constrained tree approach (see Methods for details). A constrained tree was created (Additional file 11) in which all species were combined into four major clades in accord with the overall organismal phylogeny, namely, bacteria, protists, fungi and animals. Inside the animal branch, with *Trichoplax *at the root, the amphioxus branch was placed as the sister group of vertebrates, and the segmented worms and mussel were positioned between chordates and *Trichoplax*. The fungal branch was placed as the sister group of animals. The Approximately Unbiased test result (p-value 0.148) for the tree generated with this approach (Additional files 12 and 13) shows that the "classical" constrained tree topology could not be rejected with confidence although the likelihood of that topology is much lower than that of the best tree shown in Figure 2.

The results of the phylogenetic analysis of the AANATs emphasize the major distinction between the vertebrate forms and those from other animals, presumably, owing to a dramatic acceleration of evolution at the base of the vertebrate branch. Beyond that conclusion, however, the results were quite uncertain due to the low information content of the alignment, between-branch differences in the evolutionary rates, and uneven sampling of different groups.

### Expression and characterization of bfAANATα protein

A comparison of the predicted amino acid sequences derived from the published genomic sequence and from our cloned *bfAANATα *(GenBank accession number EU380676) revealed differences in five amino acid residues scattered throughout (Additional file 4). These differences, most likely, reflect the high degree of polymorphism in *B. floridae *[18]. The effects of these differences are unknown.

**Figure 3:**
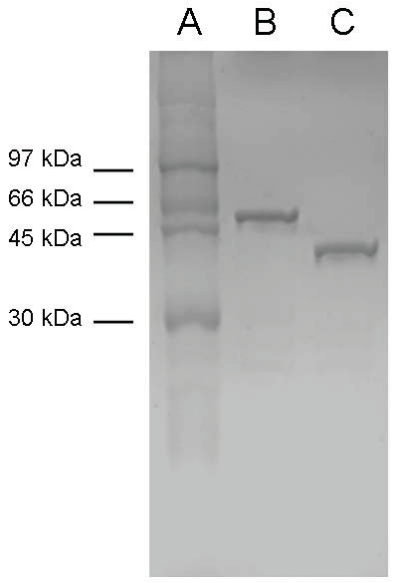
**Characterization of the recombinant bfAANAT proteins**. SDS-PAGE analysis of purified GST-bfAANAT α (B) and GST-bfAANATδ' (C), together with the Rainbow™ marker (A). Predicted relative molecular weights are 54,000 for GST-bfAANATα and 46,000 for GST-bfAANATδ'.

To determine the molecular mass of expressed bfAANATα, the GST-bfAANATα fusion protein was cleaved and bfAANATα was purified as described in the Methods. The final preparation contained a single major band of protein which migrated at ~35 kDa on SDS-PAGE (Figure 3). MALDI-TOF analysis revealed the mass to be 28515, corresponding to the predicted mass based on amino acid composition. Using the same procedure we also expressed GST-bfAANATδ'.

To establish whether the expressed protein was correctly folded, we measured binding of a fluorescent bisubstrate inhibitor of the expressed protein, CoA-HNE; binding of the inhibitor to oAANAT quenches fluorescence [15]. Here we found that bfAANATα quenched inhibitor fluorescence and that the expressed protein bound the inhibitor stoichiometrically (1:1; Figure 4A). Moreover, binding was totally disrupted by treatment with 4 M guanidine. Accordingly, the purified preparation of bfAANATα is not denatured and possesses characteristics generally similar to those of oAANAT, as regards binding of CoA-HNE.

**Figure 4:**
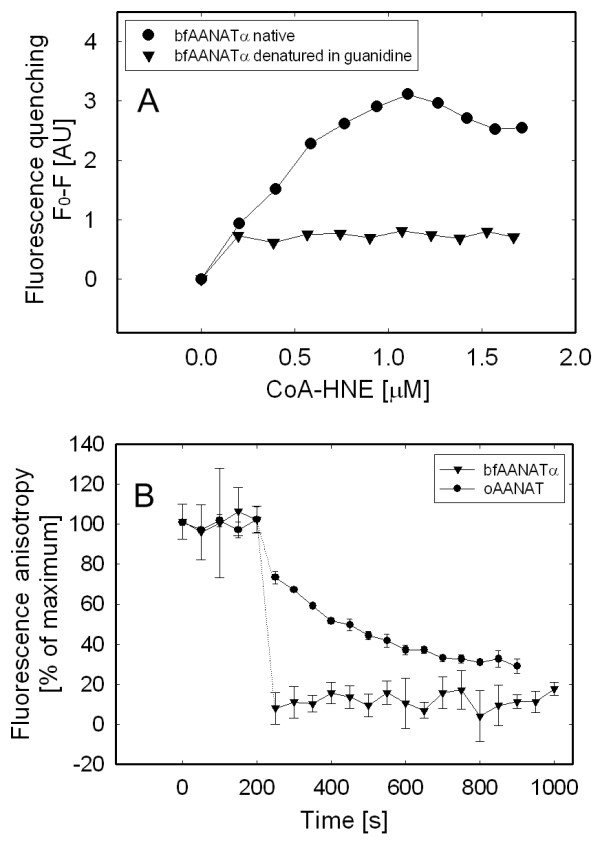
**Determination of CoA-HNE binding and off-rate**. A. Quenching of tryptophan fluorescence as a function of the binding of inhibitor CoA-HNE by recombinant GST-bfAANATα. Binding was not observed in the presence of 4 M guanidine, which denatures the protein. B. Off-rate of CoA-HNE from the active site of the enzyme was measured by displacement with another inhibitor. Fluorescence anisotropy is shown as a function of time. Samples of proteins (2 μM) were 90% saturated with CoA-HNE. After 5 min the fluorescence anisotropy was measured. At t = 200 s, CoA-T was added to a final concentration of 170 μM and fluorescence anisotropy measurement was resumed. oAANAT data from a previous study are included for comparison [15]. Data are presented as the mean ± S.E.M (N = 3). For further details see Methods.

To further characterize bfAANATα, off-rate analysis was done using the displacement strategy described previously [15], in which bound CoA-HNE is displaced by CoA-T, a non-fluorescent AANAT inhibitor [36] that does not interfere with detection of CoA-HNE (Figure 4B). This result shows that the off-rate observed for the CoA-HNE is > 0.05 s^-1^, similar to that of yeast AANAT; these rates are much faster than that for oAANAT [15], consistent with the view that the proline-containing tripeptide in the floppy loop of vertebrate AANAT enhances binding of substrate.

### Biochemical characterization of bfAANATα and bfAANATδ'

**Figure 5:**
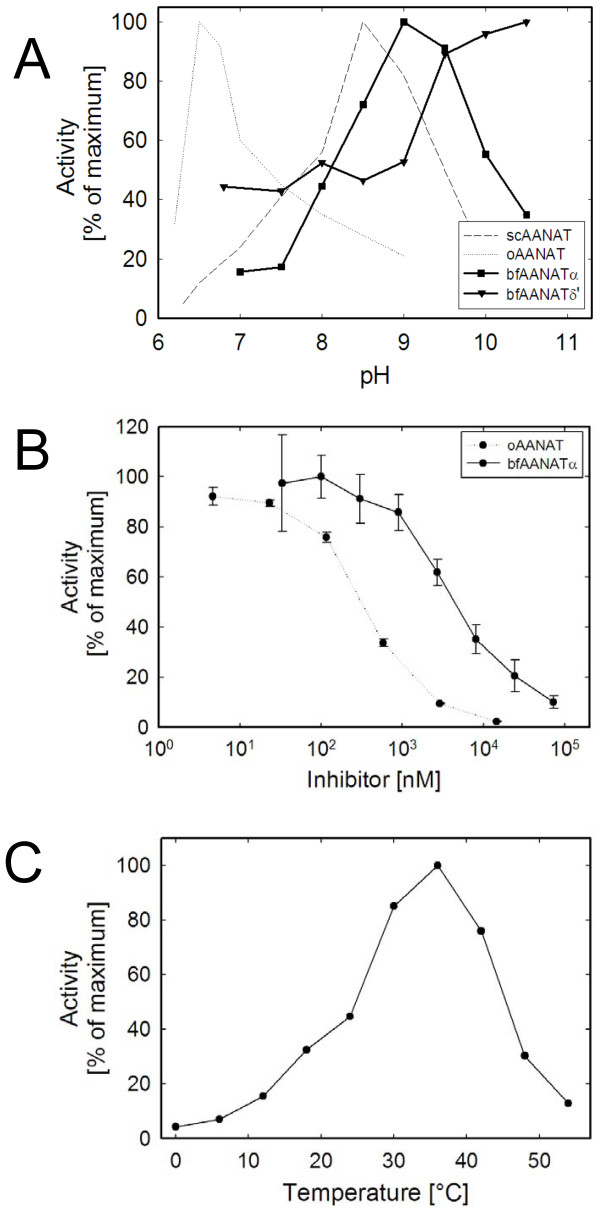
**Enzyme activity of GST-bfAANATα and GST-bfAANATδ'**. A. pH optimum. Data are presented as the percent of maximal activity for each protein. Data represent results of two independent experiments with the difference of means < 5%. For comparison, data for ovine and yeast AANAT from a previous study are shown [8]. B. Inhibition of GST-bfAANATα by bisubstrate inhibitor CoA-T. C. Acetylation activity of GST-bfAANATα towards tryptamine measured at different temperatures to determine the optimum. Data represent results of two independent experiments with the difference of means < 5%. Data in B are presented as the mean ± S.E.M (N = 3). For further details see Methods.

Activity as a function of pH: GST-bfAANATα and GST-bfAANATδ' both exhibited a pH activity profile that differed from that of ovine AANAT and yeast AANAT as examined using 10 mM tryptamine as a substrate (Figure 5A). The pH optimum for GST-bfAANATα was determined to be 9.0, which differs substantially from published data on fish, ovine and yeast AANAT (6.1, 6.5 and 8.5, respectively) [8,42]. The pH optimum for GST-bfAANATδ' seems to be even higher. Similar results were obtained using butylamine as a substrate (data not shown).

Effect of AANAT bisubstrate inhibitor: Enzyme activity was inhibited by the bisubstrate inhibitor CoA-T [36] (Figure 5B). The IC_50 _value (3 μM) was found to be ~10-fold greater than that reported for ovine AANAT [15].

Temperature dependence: The temperature optimum for bfAANATα was found to be 36°C (Figure 5C). This is similar to that of ovine AANAT (37°C), yeast AANAT (42°C) or pike AANAT-1 (37°C) [8,11].

**Figure 6:**
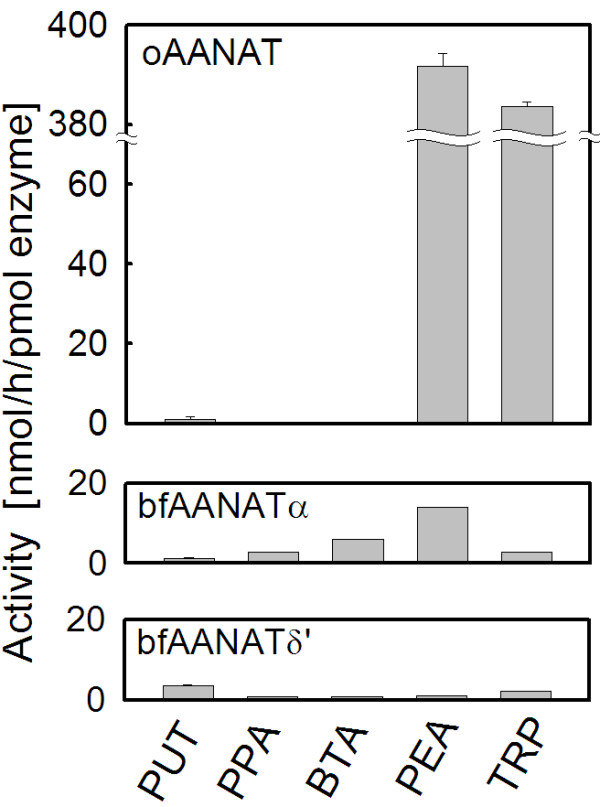
**Comparison of acetylation activity of expressed vertebrate and amphioxus AANATs**. Acetylation activity of GST-bfAANATα, GST-bfAANATδ' (both at pH 9.0) and GST-oAANAT (at pH 6.8) towards various substrates (10 mM) was determined. PUT: putrescine, PPA: propylamine, BTA: butylamine, PEA: phenylethylamine, TRP: tryptamine. Data are presented as the mean ± S.E.M (N = 3). For further details see Methods.

**Figure 7:**
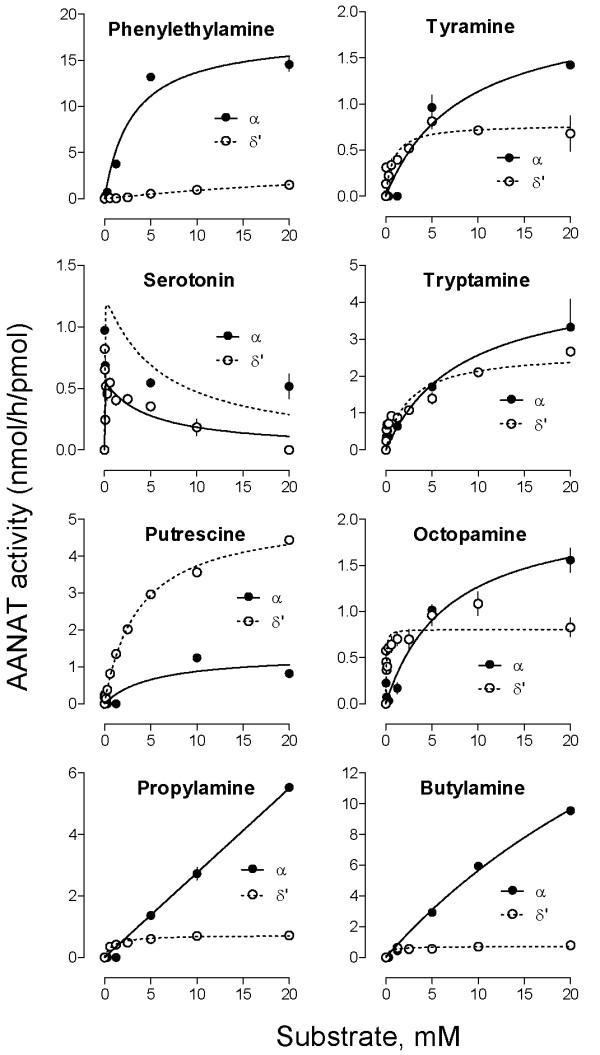
**Enzyme activity of GST-bfAANATα and GST-bfAANATδ' as a function of substrate concentration**. The enzyme assay with GST-bfAANATα and bfAANATδ' was measured at pH 9.0 and was performed as described in the Methods. Calculated values of V_max _and K_m _are given in Table 1. Data are presented as the mean ± S.E.M (N = 3).

Substrate preference: The relative capacities of oAANAT, bfAANATα and bfAANATδ' to acetylate a diamine (putrescine), alkylamines (butylamine and propylamine) and arylalkylamines (PEA and tryptamine) were determined at pH 6.8 for oAANAT, and 9.0 for bfAANATα and bfAANATδ' (Figure 6) using GST-fusion proteins. The activity of bfAANATs towards the substrates tested (10 mM) was relatively similar and fell within a 5-fold range of activity; the highest activity was observed with PEA, however, bfAANATα and bfAANATδ' seemed to show a slight preference for hydrophobic or polar substrates, respectively. The K_m _and V_max _values were determined for five biogenic arylalkylamines: octopamine, PEA, tyramine, tryptamine and serotonin (Figure 7, Table 1).

**Table 1:** Kinetic characteristics of GST-bfAANAT and GST-bfAANAT ' for selected amines.

	Substrate							
	**Phenylethylamine**		**Tyramine**		**Serotonin**		**Tryptamine**	

**bfAANAT**	**α**	**δ'**	**α**	**δ'**	**α**	**δ'**	**α**	**δ'**

**V**_**max**_	17.8 ± 1.1	4.9 ± 3	2.06 ± 0.28	0.78 ± .06	1.60 ± 0.1	0.56 ± 0.06	4.7 ± 0.8	2.7 ± 0.25

**K**_**m**_	3 ± 0.6	45 ± 36	8.2 ± 2.70	0.80 ± 0.28	0.01 ± 0.01	<0.01	8.1 ± 3.6	2.5 ± 0.7

	**Substrate**							

	**Octopamine**		**Propylamine**		**Butylamine**		**Putrescine**	

**bfAANAT**	**α**	**δ'**	**α**	**δ'**	**α**	**δ'**	**α**	**δ'**

**V**_**max**_	2.1 ± 0.2	0.80 ± 0.05	>10	0.73 ± 0.07	32.5 ± 4.1	0.7 ± 0.05	1.34 ± 0.32	5.11 ± 0.11

**K**_**m**_	6.8 ± 1.9	0.05 ± 0.01	>20	0.90 ± 0.37	47.5 ± 8.0	0.37 ± 0.23	5.2 ± 4.0	3.68 ± 0.22

Activity was measured at pH 9.0. Values are calculated from data in Figure 7. **V**_**max **_values are nmol/h/pmol enzyme and are given as the mean ± S.E.M. (N = 3 to 6). For further details see Methods.

In contrast to the relatively broad substrate preference profiles of bfAANATs observed with the substrates tested, oAANAT exhibited markedly higher activity towards the two indolamines, tryptamine and serotonin, and lower activity towards putrescine, propylamine and butylamine. This points to a marked difference in substrate preference: bfAANATα and bfAANATδ' exhibit a broad capacity to acetylate amines, including arylalkylamines and a diamine, whereas oAANAT exhibits a distinct preference for arylalkylamines with an aromatic group, including serotonin, PEA and tryptamine.

### Control of the abundance of blAANAT transcripts

In some vertebrate species, the nocturnal increase in AANAT activity is accompanied by a ~100- fold increase in the abundance of transcripts [7]. Here we used qPCR to determine whether the abundance of blAANAT mRNAs varied on a night/day basis (Additional file 14). However statistically significant differences were only observed for blAANATα. Accordingly, although it does not appear that there are marked night/day differences in expression of blAANATs, it is clear that small differences do occur.

### Analysis of tissue expression by RT-PCR

**Figure 8:**
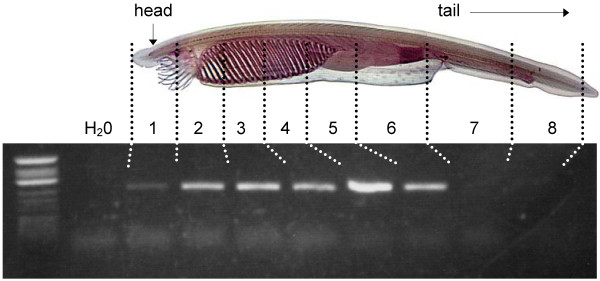
**RT-PCR localization of *blAANATα *expression**. The PCR conditions were as described in the Methods with the forward and reverse RT-PCR primers given in Additional file 1. Note that this PCR was obtained with sexually active *B. lanceolatum *(*i.e*., parts 2 to 6 contained eggs).

Investigation of tissue expression of blAANATα by RT-PCR revealed the presence of mRNA along the whole body (Figure 8). The identity of the product was confirmed by sequencing (GenBank accession number EU635724; Additional file 4).

### Daily changes in blAANAT activity

Homogenates of the heads of *B. lanceolatum *were used for measurement of AANAT activity, using 10 mM PEA as a substrate; CoA-T was added to determine the contribution from AANAT to total acetyltransferase activity. In all conditions tested, activity was significantly higher in heads sampled at midday than in those sampled at midnight (Table 2). Activity measured in the absence of added substrate was more than 50% as high as that measured in the presence of exogenous substrate (PEA); in both cases, activity was nearly completely inhibited by CoA-T, indicating that blAANAT acetylated endogenous substrates during the assay. In other studies this rhythm was confirmed, however a similar daily change was not observed in animals housed in constant darkness or constant lighting (Table 3). A detailed graph describing the inhibition of acetylation activity in homogenates by CoA-T is shown in Additional file 15.

**Table 2:** Acetylation activity measured in tissue homogenates from heads of *B. lanceolatum*

		blAANAT activity (nmol/h/μg of total protein)		
**Time of day**	**pH**	**Endogenous substrate**	**Exogenous + endogenous** **substrates**	**Exogenous + endogenous** **substrates** **+ CoA-T**

**Midday**	8.5	0.26 ± 0.15	0.68 ± 0.27	0.004 ± 0.002
		
**Midnight**		0.08 ± 0.08	0.05 ± 0.05	0.001 ± 0.001

**Midday**	9.5	0.29 ± 0.08	0.47 ± 0.17	0.004 ± 0.001
		
**Midnight**		Not detectable	Not detectable	Not detectable

Comparison of samples collected during day and night. The exogenous substrate is 10 mM PEA; 1 mM CoA-T has been used for inhibition. Data are given as the mean ± S.E.M. (N = 3 to 6).

**Table 3:** Acetylation activity in different light/darkness cycles

Lighting Conditions	blAANAT activity at pH 8.5 (nmol/h/μg of total protein)	
	
	Midday	Midnight
LD	0.299 ± 0.046	0.117 ± 0.046

LL	0.234 ± 0.063	0.256 ± 0.045

DD	0.271 ± 0.028	0. 354 ± 0.032

Acetylation activity under normal light/darkness changes (LD) compared with constant light (LL) and constant darkness (DD) conditions. The pH of the assay was 8.5; data are given as the mean ± S.E.M. (N = 3 to 6).

### *In toto *hybridization

**Figure 9:**
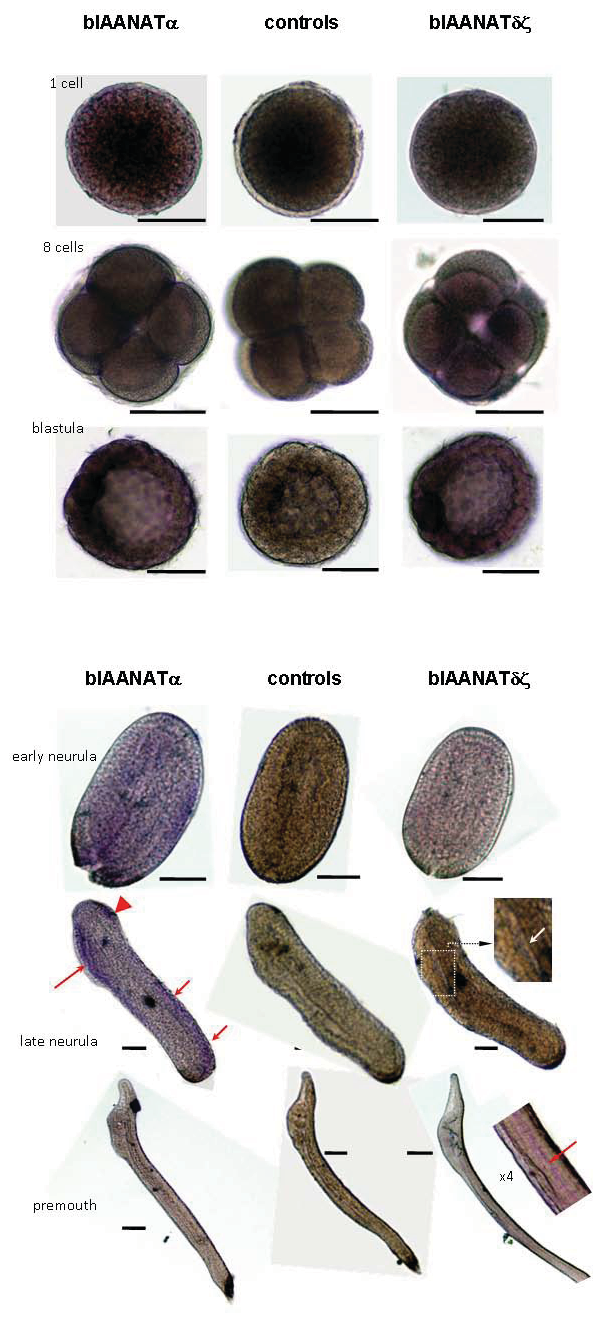
***In toto *hybridization of *B. lanceolatum***. Eggs and embryos were hybridized as indicated in the Methods section either with the α subtype (left column) or the δζ subtype (right column) probes. The central column in the image presents the result of a typical control obtained using a sense probe. In these studies, both α and δζ sense probes were used; neither produced labeling. Bar = 50 μm. For further details see Methods.

Expression of one or both *blAANAT*s was detected by *in toto *hybridization at all stages examined, in varying patterns. Although it is difficult to quantify the colorimetric reactions, it seemed that the α form was more highly expressed than the δζ form at early stages, whereas the opposite seemed true at later stages. The first specific hybridization signal was detected at the 8-cell embryo and was stronger in 4 out of the 8 cells. At the blastula stage most of the cells exhibited a diffuse labeling with both probes; however, a group of cells exhibited a more intense dark signal (Figure 9, left side). At the early neurula stage, the labeling appeared diffuse and weak with the δζ probe, whereas some regionalization appeared with the α probe. This tendency was even more apparent at the late neurula stage where the α probe gave a specific labeling in the antero-dorsal (head) and posterio-dorsal parts of the embryo, as well as antero-ventrally in the general area of the intestine. At the premouth stage, no labeling with the α probe was detected, whereas the δζ probe hybridized from head to tail in a dorsal band, corresponding to the neural tube.

## Discussion

The publication of the near complete amphioxus genome has marked a new era in understanding chordate evolution [18]. The studies presented in this report, which were made possible by this advance, focus on one family of genes and extends our understanding of this gene family in amphioxus and, potentially, in animals in general.

### AANATs in the amphioxus genome and evolution of the AANAT family

Analysis of the amphioxus genome indicates that it lacks a vertebrate-like *AANAT*. In contrast, our analysis shows that the amphioxus genome contains a set of closely related *AANAT *homologs that are similar in their gene and protein architectures to homologs from invertebrates, protists, fungi, and bacteria. None of these non-vertebrate homologs possesses the defining structural features of vertebrate *AANATs*.

The discovery of the family of AANATs in amphioxus sheds light on the timing of the emergence of the typical vertebrate AANATs that, most likely, occurred subsequent to the radiation of the vertebrate lineage from the common ancestor with cephalochordates. It appears that the change in the *AANAT *gene architecture at the base of the vertebrate tree was accompanied by a substantial acceleration of the evolutionary rate which is the likely reason why the amphioxus AANAT homologs are more similar to those from fungi and protists than to those from vertebrates. The results of phylogenetic analysis of the AANAT family presented here were not entirely conclusive owing, in part, to the relatively small number of phylogenetically informative positions in the multiple sequence alignment of the AANAT family, and in part, to the non-uniformity of evolutionary rates, and perhaps, to a rapid divergence associated with evolutionary transitions. The best ML (maximum likelihood) tree showed multiple deviations from the species tree but the topology corresponding to the species tree, including the cephalochordate-vertebrate clade, could not be rigorously rejected. Thus, the phylogeny of AANATs might follow the organismal phylogeny of animals, taking into account the apparent multiple losses, e.g., in arthropods, nematodes, and echinodermata, as well as accelerations of evolution, particularly, in vertebrates. Previous analyses of the phyletic patterns and phylogenies of the AANATs led to the suggestion that horizontal gene transfer, perhaps, even into the animal lineage or the animal-fungal ancestral lineage was a major contribution to the evolution of this family [43]. That possibility still remains considering the patchy phyletic distribution of the AANATs and the irregularities in the topologies of the phylogenetic trees, such as the polyphyly of fungi and the unusual assortment of protists. However, given the lack of strong resolution in the trees, the major changes in evolutionary rates like the one in vertebrates, and the extreme propensity of the *AANAT *family for gene loss, scenarios that involve unusual routes of horizontal transfer should be addressed with much caution.

### Biochemical features of bfAANATα and bfAANATδ'

The biochemical characteristics of bfAANATα and bfAANATδ' were analyzed in this study. Both appear to have significantly lower intrinsic activity at pH 6.8 as compared to oAANAT. The substrate preference spectrum of the bfAANATs is broader than that of oAANAT. As with other non-vertebrate AANATs, these differences are likely to reflect differences in the primary structure of the enzyme, including the presence in vertebrate AANAT of two histidines near the site of acetyl transfer. These are thought to be part of a proton wire which accelerates catalysis by facilitating the expulsion of protons generated during acetyl transfer, thereby preventing their accumulation and the resulting acidification of the active site (7).

Another important difference in the primary structure between vertebrate and amphioxus AANATs occurs in a floppy loop which is critical for catalysis, apparently by increasing the dwell time of amine stubstrates in the binding pocket [15]. The floppy loop of the vertebrate AANATs is three residues longer than that of known AANAT homologs, including amphioxus. This addition appears to enhance catalysis by directing the movement of the floppy loop and preventing intramolecular interactions which inhibit binding. As a result, the off-rate of substrate is substantially increased when these three residues are experimentally deleted [15]. During the course of evolution these three residues were added and, as shown here, this was associated with a decrease in the off-rate relative to that of amphioxus and yeast AANATs [15].

### Regional expression of blAANAT

Our studies revealed that *blAANATα *is expressed throughout the body (Figure 8), in contrast to the pattern of expression of vertebrate *AANAT*, which is primarily limited to structures located in the head: pineal gland, retina and in some cases the pituitary gland [44]. The development of these structures is strongly dependent on expression of the transcription factor Pax6. Pax6 expression in amphioxus is limited to the frontal organ and lamellar organ; the latter has been identified as a possible pineal precursor, based on its medial location and presence of photoreceptors in the tissue [45].

### *In toto *hybridization

The *in toto *hybridization analyses revealed expression of both α and δζ genes early in development in *B. lanceolatum*. It seems that the relative strength of expression changes during development. blAANATδζ was found to be expressed in a region close to the neural tube. This observation and the finding that these bfAANATs acetylate the biogenic amines serotonin and octopamine (Table 1, Figure 7), suggest to us that it is reasonable to consider that these enzymes play a role in the metabolism of these neurotransmitters.

### The possible roles of amphioxus AANATs

The unexpected finding that several of the seven *AANATs *in the amphioxus genome are expressed is in sharp contrast to the situation in vertebrates, most of which contain only a single *AANAT*. The existence of multiple *bfAANAT *genes seems to be the product of several duplications of this gene because there is no evidence of genome duplication events within the amphioxus line. Thus, the *AANATs *of amphioxus represents a typical lineage-specific expansion of a paralogous gene family, a phenomenon that is widely observed in eukaryotes and is generally thought to reflect specific physiological adaptations [46]. The lineage-specific expansion of *AANAT*s in amphioxus is the most extensive so far detected for this family but smaller expansions are seen in *Trichoplax*, as demonstrated here, and in teleost fish. The finding that the amino acid sequences of some of the paralogous amphioxus AANATs are less than 40% identical suggests distinctly different substrate preferences, stability and kinetic characteristics.

Based on these findings, it seems reasonable to hypothesize that each of the bfAANAT paralogs plays a unique biological role. This function is obviously not the time keeping by melatonin synthesis as it is in vertebrates, since there is no circadian rhythm in the blAANAT activity; moreover, no melatonin has been found in amphioxus (our unpublished observation), and attempts to identify melatonin receptors in amphioxus were unsuccessful [47]. One likely function of bfAANATs is detoxification. As discussed above, this possibility is supported by the observation that bfAANATα and bfAANATδ' acetylate diamines and alkylamines, in addition to arylalkylamines. Amphioxus lives buried in sediment, and, under these conditions, a wide range of AANAT substrates might be generated as a result of biodegradation. These molecules could be nonspecifically ingested or otherwise absorbed by the organism and as a result might represent a challenge to amphioxus physiology. Under these circumstances, the presence of multiple copies of *AANAT *would provide a critical survival advantage. Considering the similarity between the life styles of amphioxus and *Trichoplax*, it is tempting to speculate that these animals experience similar pressures from toxic environmental molecules, driving the fixation of gene duplications in the *AANAT *family. It is also possible that the functions of one or more bfAANATs evolved from detoxification and pH regulation to a more specialized role in neurobiology. This suggestion is based on the evidence that demonstrated substrates of bfAANATα and bfAANATδ', serotonin and octopamine, are thought to play a role in neurotransmission in amphioxus [48,49]. The acetylation of these compounds by bfAANATs is likely to inactivate them, thereby contributing to the process of neurotransmission by termination of neural stimulation. Transmitter inactivation is essential for efficient neural signaling.

In closing, it should be emphasized that the study presented here characterized only two of the seven bfAANAT proteins. Accordingly, it is possible that other family members have distinctly different substrate preferences and unique biological roles.

### The implications of day/night changes in AANAT activity

We found a daily rhythm in total AANAT activity, with high values during the day. The finding that a daily rhythm in head AANAT activity did not persist under constant light or constant darkness conditions is consistent with the interpretation that the AANAT rhythm is not driven by a circadian clock, such as that found to operate in the amphioxus head [50].

## Conclusions

The unexpected finding of multiple *bfAANAT *homolog genes points to the importance of this gene family in amphioxus. The function of these genes appears to be detoxification of exogenous and endogenous amines, the latter including neurotransmitters. It will be of interest to determine the precise distribution of each gene, how their expression is regulated and how each gene contributes to amphioxus biology.

## Abbreviations

AANAT: arylalkylamine *N*-acetyltransferase; oAANAT: ovine AANAT; scAANAT: *Saccharomyces cerevisiae *AANAT; bfAANAT: *B. floridae *AANAT; blAANAT: *B. lanceolatum *AANAT; AcCoA: acetyl coenzyme A; AU: arbitrary unit; CoA-HNE: hydroxynaphthylethylamine coenzyme A; CoA-T: tryptamine coenzyme A; DTT: dithiothreitol; GST: glutathione *S*-transferase; PBS: phosphate buffer saline; PEA: phenylethylamine; TCEP: tris[2-carboxyethyl]phosphine.

## Authors' contributions

JP performed biochemical, physico-chemical, molecular biological studies and drafted the manuscript, SS participated in biochemical studies, LB participated in biological studies, SLC participated in molecular genetic studies and helped to draft the manuscript, JLW participated in biochemical studies, GB, PG and JF participated in biochemical and biological studies, MVO and EVK performed sequence and phylogenetic analysis and DCK conceived of the study, participated in its coordination and helped to draft the manuscript. All authors read and approved the final manuscript.

## Supplementary Material

Additional file 1 **Primers used for cloning PCR, RT-PCR localization of blAANAT expression and qPCR experiments**. Cloning primers were based on B. floridae genomic sequence and used to amplify the indicated *blAANATs*. Click here for file

Additional file 2 **Pairwise comparison scores of protein sequences**. Pairwise comparison scores based on CLUSTALW multiple sequence alignment of deduced peptide sequences of all 7 amphioxus AANAT homologs and their known alleles. Shaded boxes designate comparisons between alleles. The coordinates of each gene and allele (') in genomic assembly v1.0 are (scaffold number:sequence location): *bfAANATα*, 54:2,458,444-2,459,187; *bfAANATα'*, 56:737,209-736,466; *bfAANATβ*, 54:2,461,188-2,461,679; *bfAANATβ'*, 56:734,493-734,002; *bfAANATγ*, 54:2,488,683-2,489,174; *bfAANATγ'*, 56:702,576-702,085; *bfAANATδ*, 54:2,491,399-2,491,929; *bfAANATδ'*, 54:699,628-699,095; *bfAANATε*, 200:1,410,854-1,410,362 (includes deletion of 1 of 6 consecutive A's at bp 133 to maintain reading frame); *bfAANATζ*, 200:1,409,115-1,408,588; *bfAANATη*, 234:825,177-825,883 (includes insertion of 1 base [4^th ^A] at position 442 to maintain reading frame); *bfAANATη'*, 81:2,115,130-2,115,834 (includes insertion of 1 base [4^th ^T] at position 106 to maintain reading frame; and deletion of 1 base [4^th ^G] at position 652 to maintain reading frame). Click here for file

Additional file 3 Positioning of the 7 genes for *bfAANAT *on the assembly scaffolds as given in *B*. floridae genome assembly v2.0. Click here for file

Additional file 4 **Comparison of deduced amino acid sequences of characterized AANATs to reference genomic sequences**. A. AANATα. B. AANATδ'. (g) denotes derived from the published *B. floridae *genomic sequence. (c) denotes cloned from *B. floridae *head cDNA, and are the full length recombinant proteins used for enzyme characterization. (f) denotes a partial fragment cloned from *B. lanceolatum *head cDNA and used as a probe for *in toto *hybridization. Highlighted residues are those that differ from the published *B. floridae *genomic sequence. "--", gap inserted to preserve alignment; "...", missing sequence. Click here for file

Additional file 5 **List of species used for construction of phylogenic tree**. List of species containing AANAT homologs used for the construction of the phylogenetic tree shown in Figure 2. "gi" numbers precede the species name, where available; the source of the sequence is given in parentheses. Sequences for which "gi" numbers are not available are given in Additional file 6. Click here for file

Additional file 6 **FASTA sequences of AANAT homologs**. FASTA sequences of those of AANAT homologs used for the construction of the phylogenetic tree shown in Figure 2, but not having "gi" numbers, (as listed in Additional file 5). Click here for file

Additional file 7 **Multiple alignment used for construction of the phylogenic tree**. Multiple sequence alignment of the conserved regions of selected AANAT sequences from various species used for the construction of the phylogenetic tree in Figure 2. Species included in this alignment are given in Additional file 5. Click here for file

Additional file 8 **Average evolutionary distance (substitution per position) between major taxonomic groups for AANAT proteins based on the truncated alignment shown in Additional file****7****(JTT substitution model, gamma distribution 1).** Click here for file

Additional file 9 **Average differences (p-distance) between major taxonomic groups for AANAT proteins, based on the truncated alignment shown in Additional file****7****, calculated using the MEGA program**[**34**]. Click here for file

Additional file 10 **Average percent identity between major taxonomic groups for AANAT proteins based on the truncated alignment shown in Additional file****7****, calculated from Additional file****9**. Click here for file

Additional file 11 Model of constrained large-scale taxonomic topology tree of selected AANAT sequences. Click here for file

Additional file 12 **Topology tree - normal view**. Reconstructed constrained large-scale taxonomic topology tree of selected AANAT sequences - normal view based on model shown on Additional file 11. The scale bar represents the number of substitutions per position; numbers in parenthesis show number of species. The number at internal branches shows the bootstrap support (%). Click here for file

Additional file 13 **Topology tree - radiation view**. Reconstructed constrained large-scale taxonomic topology tree of selected AANAT sequences - radiation view based on model shown on Additional file 11. The scale bar represents the number of substitutions per position; numbers in parenthesis show number of species. The number at internal branches shows the bootstrap support (%). Click here for file

Additional file 14 **Comparison of day/night expression of three blAANATs detected by qPCR in the head and body of amphioxus**. Primers used are given in Additional file 1. Asterisk indicates statistical significance of day/night differences. Data are presented as the mean ± S.E.M. (N = 3). For further details see Methods. Click here for file

Additional file 15 **Inhibition of acetylation activity**. Inhibition of acetylation activity in homogenate from amphioxus heads at pH 8.5 using 10 mM PEA and 0.5 mM AcCoA as a substrate and various concentrations of CoA-T as an inhibitor. Data are presented as the mean ± S.E.M. (N = 3). Click here for file

## References

[B1] Dyda F Klein DC Hickman AB GCN5-related N-acetyltransferases: a structural overview Annu Rev Biophys Biomol Struct 2000 29 81 103 10.1146/annurev.biophys.29.1.81 10940244 PMC4782277

[B2] Coon SL Klein DC Evolution of arylalkylamine N-acetyltransferase: emergence and divergenceMol Cell Endocrinol Mol Cell Endocrinol 2006 252 2 10 10.1016/j.mce.2006.03.039 16697105 PMC1578506

[B3] Vetting MWS de Carvalho LP Yu M Hegde SS Magnet S Roderick SL Blanchard JS Structure and functions of the GNAT superfamily of acetyltransferases Arch Biochem Biophys 2005 433 212 226 10.1016/j.abb.2004.09.003 15581578

[B4] Klein DC Coon SL Roseboom PH Weller JL Bernard M Gastel JA Zatz M Iuvone PM Rodriguez IR Begay V Falcon J Cahill GM Cassone VM Baler R The melatonin rhythm-generating enzyme: molecular regulation of serotonin N-acetyltransferase in the pineal gland Recent Prog Horm Res 1997 52 307 357 9238858

[B5] Coon SL Roseboom PH Baler R Weller JL Namboodiri MA Koonin EV Klein DC Pineal serotonin N-acetyltransferase: expression cloning and molecular analysis Science 1995 270 1681 1683 10.1126/science.270.5242.1681 7502081

[B6] Arendt J Melatonin and the Mammalian Pineal Gland 1995 London: Chapman and Hall

[B7] Klein DC Arylalkylamine N-acetyltransferase: "the Timezyme" J Biol Chem 2007 282 4233 4237 10.1074/jbc.R600036200 17164235

[B8] Ganguly S Mummaneni P Steinbach PJ Klein DC Coon SL Characterization of the Saccharomyces cerevisiae homolog of the melatonin rhythm enzyme arylalkylamine N-acetyltransferase (EC 2.3.1.87) J Biol Chem 2001 276 47239 47247 10.1074/jbc.M107222200 11559708

[B9] Liu B Sutton A Sternglanz R A yeast polyamine acetyltransferase J Biol Chem 2005 280 16659 16664 10.1074/jbc.M414008200 15723835

[B10] Begay V Falcon J Cahill GM Klein DC Coon SL Transcripts encoding two melatonin synthesis enzymes in the teleost pineal organ: circadian regulation in pike and zebrafish, but not in trout Endocrinology 1998 139 905 912 10.1210/en.139.3.905 9492019

[B11] Coon SL Begay V Deurloo D Falcon J Klein DC Two arylalkylamine N-acetyltransferase genes mediate melatonin synthesis in fish J Biol Chem 1999 274 9076 9082 10.1074/jbc.274.13.9076 10085157

[B12] Falcon J Gothilf Y Coon SL Boeuf G Klein DC Genetic, temporal and developmental differences between melatonin rhythm generating systems in the teleost fish pineal organ and retina J Neuroendocrinol 2003 14 378 382 10.1046/j.1365-2826.2003.00993.x 12622837

[B13] Zilberman-Peled B Benhar I Coon SL Ron B Gothilf Y Duality of serotonin-N-acetyltransferase in the gilthead seabream (Sparus aurata): molecular cloning and characterization of recombinant enzymes Gen Comp Endocrinol 2004 138 139 147 10.1016/j.ygcen.2004.05.007 15302263

[B14] Scheibner KA De Angelis AJ Burley SK Cole PA Investigation of the roles of catalytic residues in serotonin N-acetyltransferase J Biol Chem 2002 277 18118 18126 10.1074/jbc.M200595200 11884405

[B15] Pavlicek J Coon SL Ganguly S Weller JL Hassan SA Sackett DL Klein DC Evidence that proline focuses movement of the floppy loop of arylalkylamine N-acetyltransferase (EC 2.3.1.87) J Biol Chem 2008 283 14552 14558 10.1074/jbc.M800593200 18362150 PMC2386931

[B16] Iyer LM Aravind L Coon SL Klein DC Koonin EV Evolution of cell-cell signaling in animals: did late horizontal gene transfer from bacteria have a role? Trends Genet 2004 20 292 299 10.1016/j.tig.2004.05.007 15219393

[B17] Fuentes M Schubert M Dalfo D Candiani S Benito E Gardenyes J Godoy L Moret F Illas M Patten I Permanyer J Oliveri D Boeuf G Falcon J Pestarino M Fernandez JG Albalat R Laudet V Vernier P Escriva H Preliminary observations on the spawning conditions of the European amphioxus (Branchiostoma lanceolatum) in captivity J Exp Zoolog B Mol Dev Evol 2004 302 384 391 10.1002/jez.b.20025 15287102

[B18] Putnam NH Butts T Ferrier DE Furlong RF Hellsten U Kawashima T Robinson-Rechavi M Shoguchi E Terry A Yu JK Benito-Gutiérrez EL Dubchak I Garcia-Fernàndez J Gibson-Brown JJ Grigoriev IV Horton AC de Jong PJ Jurka J Kapitonov VV Kohara Y Kuroki Y Lindquist E Lucas S Osoegawa K Pennacchio LA Salamov AA Satou Y Sauka-Spengler T Schmutz J Shin-I T Toyoda A Bronner-Fraser M Fujiyama A Holland LZ Holland PW Satoh N Rokhsar DS The amphioxus genome and the evolution of the chordate karyotype Nature 2008 453 1064 1071 10.1038/nature06967 18563158

[B19] Altschul SF Madden TL Schäffer AA Zhang J Zhang Z Miller W Lipman DJ Gapped BLAST and PSI-BLAST: a new generation of protein database search programs Nucleic Acids Res 1997 25 3389 3402 10.1093/nar/25.17.3389 9254694 PMC146917

[B20] Edgar RC MUSCLE: multiple sequence alignment with high accuracy and high throughput Nucleic Acids Res 2004 32 1792 1797 10.1093/nar/gkh340 15034147 PMC390337

[B21] Jobb G von Haeseler A Strimmer K TREEFINDER: a powerful graphical analysis environment for molecular phylogenetics BMC Evol Biol 2004 4 18 10.1186/1471-2148-4-18 15222900 PMC459214

[B22] Whelan S Goldman N A general empirical model of protein evolution derived from multiple protein families using a maximum-likelihood approach Mol Biol Evol 2001 18 691 699 11319253 10.1093/oxfordjournals.molbev.a003851

[B23] Jones DT Taylor WR Thornton JM The rapid generation of mutation data matrices from protein sequences Comput Appl Biosci 1992 8 275 282 1633570 10.1093/bioinformatics/8.3.275

[B24] Müller T Vingron M Modeling amino acid replacement J Comput Biol 2000 7 761 776 10.1089/10665270050514918 11382360

[B25] Henikoff S Henikoff JG Amino acid substitution matrices from protein blocks Proc Natl Acad Sci USA 1992 89 10915 10919 10.1073/pnas.89.22.10915 1438297 PMC50453

[B26] Kosiol C Goldman N Different versions of the Dayhoff rate matrix Mol Biol Evol 2005 22 193 199 10.1093/molbev/msi005 15483331

[B27] Adachi J Waddell PJ Martin W Hasegawa M Plastid genome phylogeny and a model of amino acid substitution for proteins encoded by chloroplast DNA J Mol Evol 2000 50 348 358 10795826 10.1007/s002399910038

[B28] Dimmic MW Rest JS Mindell DP Goldstein RA rtREV: an amino acid substitution matrix for inference of retrovirus and reverse transcriptase phylogeny J Mol Evol 2002 55 65 73 10.1007/s00239-001-2304-y 12165843

[B29] Veerassamy S Smith A Tillier ER A transition probability model for amino acid substitutions from blocks J Comput Biol 2003 10 997 1010 10.1089/106652703322756195 14980022

[B30] Strimmer K Rambaut A Inferring confidence sets of possibly misspecified trees Proc Biol Sci 2002 269 137 142 10.1098/rspb.2001.1862 11798428 PMC1690879

[B31] Page RD TreeView: an application to display phylogenetic trees on personal computers Comput Appl Biosci 1996 12 357 358 8902363 10.1093/bioinformatics/12.4.357

[B32] Shimodaira H An approximately unbiased test of phylogenetic tree selection Syst Biol 2002 51 492 508 10.1080/10635150290069913 12079646

[B33] Felsenstein J Inferring phylogenies from protein sequences by parsimony, distance, and likelihood methods Methods Enzymol 1996 266 418 427 full_text 8743697 10.1016/s0076-6879(96)66026-1

[B34] Kumar S Tamura K Nei M MEGA3: Integrated software for Molecular Evolutionary Genetics Analysis and sequence alignment Briefings in Bioinformatics 2004 2 150 163 10.1093/bib/5.2.150 15260895

[B35] De Angelis J Gastel J Klein DC Cole PA Kinetic analysis of the catalytic mechanism of serotonin N-acetyltransferase (EC 2.3.1.87) J Biol Chem 1998 273 3045 3050 10.1074/jbc.273.5.3045 9446620

[B36] Khalil EM Cole PA A potent inhibitor of the melatonin rhythm enzyme J Am Chem Soc 1998 120 6195 6196 10.1021/ja981365a

[B37] Laemmli UK Cleavage of structural proteins during the assembly of the head of bacteriophage T4 Nature 1970 227 680 685 10.1038/227680a0 5432063

[B38] Fuentès M Benito E Bertrand S Paris M Mignardot A Godoy L Jimenez-Delgad S Oliveri D Candiani S Hirsinger E D'Aniello S Pascual-Anaya J Maeso J Pestarino M Vernier P Nicolas JF Schubert M Laudet V Geneviere AM Albalat R Fernandez JG Holland ND Escriva H Insights into spawning behavior and development of the European amphioxus (Branchiostoma lanceolatum) J Exp Zool B-Mol Dev Evol 2007 308B 484 494 10.1002/jez.b.21179 17520703

[B39] Holland LZ Holland PWH Holland ND Ferraris JD, Palumbi SR Revealing homologies between body parts of distantly related animals by in situ hybridization to developmental genes: Amphioxus vs. vertebrates Molecular Zoology: Advances, Strategies, and Protocols 1996 New York: Wiley-Liss 267 282

[B40] Besseau L Benyassi A Moller M Coon SL Weller JL Boeuf G Klein DC Falcón J Melatonin pathway: breaking the 'high-at-night' rule in trout retina Exp Eye Res 2006 82 620 627 10.1016/j.exer.2005.08.025 16289161

[B41] Keeling PJ Burger G Durnford DG Lang BF Lee RW Pearlman RE Roger AJ Gray MW The tree of eukaryotes Trends Ecol Evol 2005 20 670 676 10.1016/j.tree.2005.09.005 16701456

[B42] Benyassi A Schwartz C Coon SL Klein DC Falcon J Melatonin synthesis: arylalkylamine N-acetyltransferases in trout retina and pineal organ are different Neuroreport 2000 11 255 258 10.1097/00001756-200002070-00006 10674465

[B43] Iyer LM Aravind L Coon SL Klein DC Koonin EV Evolution of cell-cell signaling in animals: did late horizontal gene transfer from bacteria have a role? Trends Genet 2004 20 292 299 10.1016/j.tig.2004.05.007 15219393

[B44] Fleming JV Barrett P Coon SL Klein DC Morgan PJ Ovine arylalkylamine N-acetyltransferase in the pineal and pituitary glands: differences in function and regulation Endocrinology 1999 140 972 978 10.1210/en.140.2.972 9927331

[B45] Glardon S Holland LZ Gehring WJ Holland ND Isolation and developmental expression of the amphioxus Pax-6 gene (AmphiPax-6): insights into eye and photoreceptor evolution Development 1998 125 2701 2710 9636084 10.1242/dev.125.14.2701

[B46] Lespinet O Wolf YI Koonin EV Aravind L The role of lineage-specific gene family expansion in the evolution of eukaryotes Genome Res 2002 12 1048 1059 10.1101/gr.174302 12097341 PMC186617

[B47] Vernadakis AJ Bemis WE Bittman EL Localization and partial characterization of melatonin receptors in amphioxus, hagfish, lamprey, and skate Gen Comp Endocrinol 1998 110 67 78 10.1006/gcen.1997.7042 9514841

[B48] Moret F Guilland JC Coudouel S Rochette L Vernier P Distribution of tyrosine hydroxylase, dopamine, and serotonin in the central nervous system of amphioxus (Branchiostoma lanceolatum): implications for the evolution of catecholamine systems in vertebrates J Comp Neurol 2004 468 135 150 10.1002/cne.10965 14648696

[B49] Burman C Maqueira B Coadwell J Evans PD Eleven new putative aminergic G-protein coupled receptors from Amphioxus (Branchiostoma floridae): identification, sequence analysis and phylogenetic relationship Invert Neurosci 2007 7 87 98 10.1007/s10158-006-0041-z 17225134

[B50] Schomerus C Korf HW Laedtke E Moret F Zhang Q Wicht y Nocturnal behavior and rhythmic period gene expression in a lancelet, Branchiostoma lanceolatum J Biol Rhythms 2008 23 170 181 18375866 10.1177/0748730407313363

[B51] Chenna R Sugawara H Koike T Lopez R Gibson TJ Higgins DG Thompson JD Multiple sequence alignment with the Clustal series of programs Nucleic Acids Res 2003 31 3497 3500 10.1093/nar/gkg500 12824352 PMC168907

