# Supplementary material for: Evolution of AANAT: expansion of the gene family in the cephalochordate amphioxus
Source: BMC Evol Biol. 2010 May 25;10:154. doi: 10.1186/1471-2148-10-154 (PMC2897805; doi:10.1186/1471-2148-10-154)
Supplement: Additional file 1 — Primers used for cloning PCR, RT-PCR localization of blAANAT expression and qPCR experiments. Cloning primers were based on B. floridae genomic sequence and used to amplify the indicated blAANATs. [file 1471-2148-10-154-S1.PDF]

**Cloning primers**	
cAANATα F1	AAAACAAGCAGTACCCGCGC
cAANATα F2	GTCATCCGAGTGATTTGCAA
cAANATα R1	AGGTCCAGGCAACACTCGTA
cAANATα R2	GACCAAGGTGAAGCCCACCC
cAANATβ F1	ATGGCGGAAGGGAACGTACG
cAANATβ F2	ACTGGAGTMCGCTGGGTATC
cAANATβ R1	CCGTGYACAACTTCTGACAGC
cAANATβ R1	AGAACGAACCCTGCCTTGGT
cAANATγε F1	GCTGCAGTGCSTCCAAGAGGT
cAANATγε F2	CCTGCAGACGAAGCAGAACC
cAANATγε R1	ATAACTYCAGKATACATTCAT
cAANATγε R2	CTCGTGACWTATCAGACATAT
cAANATδζ F1	CCAAGAGGTTCAGCAAGCTTA
cAANATδζ F2	CCTRAAGATTATGAAGATCTG
cAANATδζ R1	ATAACTCCAGGATACATTCA
cAANATδζ R2	CATATCAGACATATCCTCTT
cAANATη F1	AAAACAATCAGAACCCACGC
cAANATη F2	GTCATCCGGGTGATTCGCAA
cAANATη R2	ACCGCGACCCGGCCTTGATC
**RT-PCR primers**	
AANAT F1	CCCCTCATCTGTGTTTCGGGTACTT
AANAT R1	AAGGTAAACCCCACCCTGGTGTAGA
**qPCR primers**	
qAANATα F1	CCAAGAGCACTACCGC
qAANATα R1	CACCCTGGTGTAGACA
qAANATγ F1	ATGAAGAGATACAGTGAG
qAANATγ R1	GTAGAACGAACTCATGGAGT

qAANATδζ F1	CCTGGATGACGGTTTACA
qAANATδζ R1	ACGAACTTATGGAGTAGTCT
qL17 F1	CAAGAATACCCGCGAAAC
qL17 R1	TGTAGGGATTGATACGGC
qL18 F1	GCGTACCAATGCCAAG
qL18 R1	ACACGAAGGCAGCAAA
qActin F1	CCTTCTACAACGAGCTGCGTATC
qActin R1	TCGTGGACACCAGTGGACTC
