# Supplementary figures and images for: Evolution of AANAT: expansion of the gene family in the cephalochordate amphioxus
Source: BMC Evol Biol. 2010 May 25;10:154. doi: 10.1186/1471-2148-10-154 (PMC2897805; doi:10.1186/1471-2148-10-154)

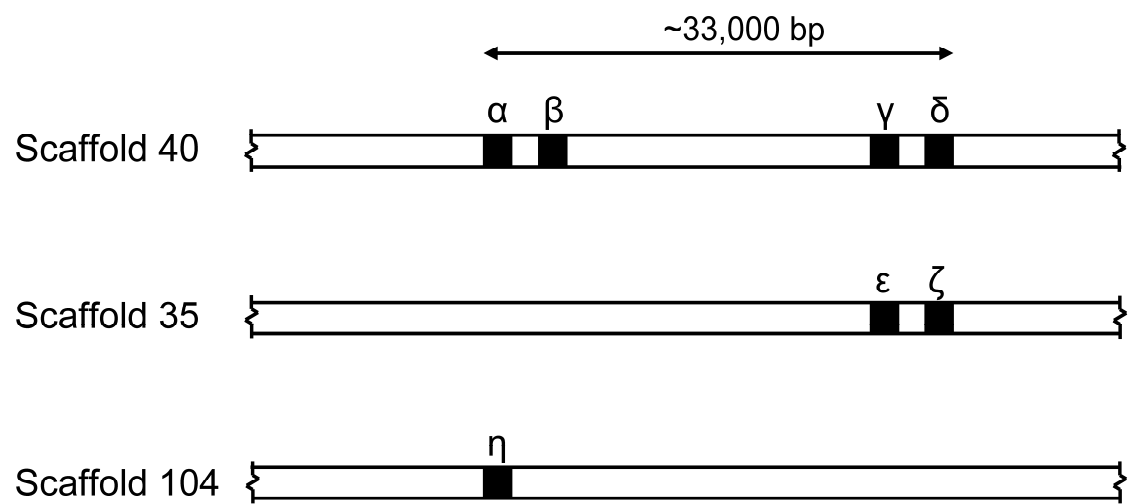

Supplement: Additional file 3 — Positioning of the 7 genes for bfAANAT on the assembly scaffolds as given in B. floridae genome assembly v2.0. [file 1471-2148-10-154-S3.PDF]

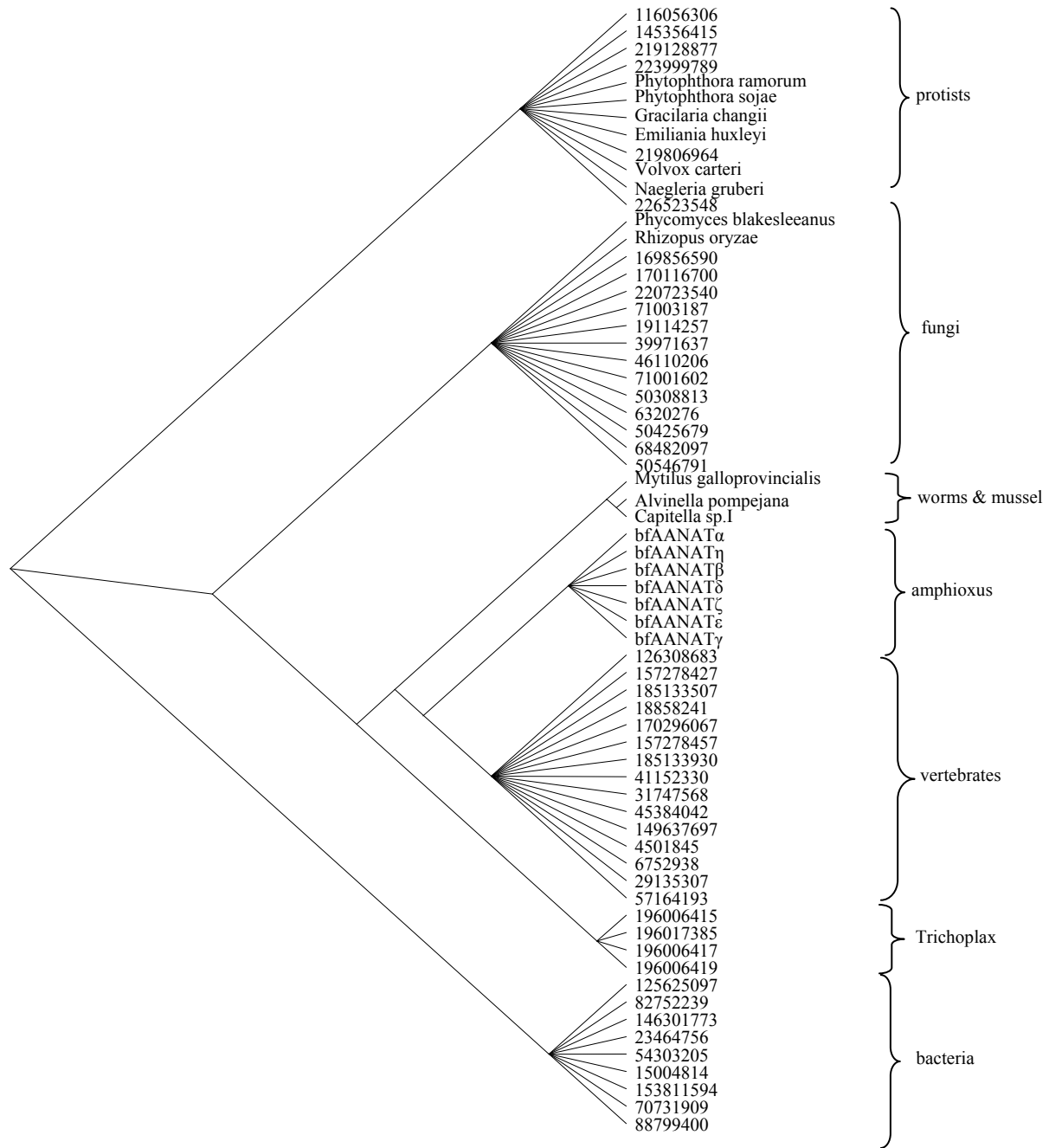

Supplement: Additional file 11 — Model of constrained large-scale taxonomic topology tree of selected AANAT sequences. [file 1471-2148-10-154-S11.PDF]

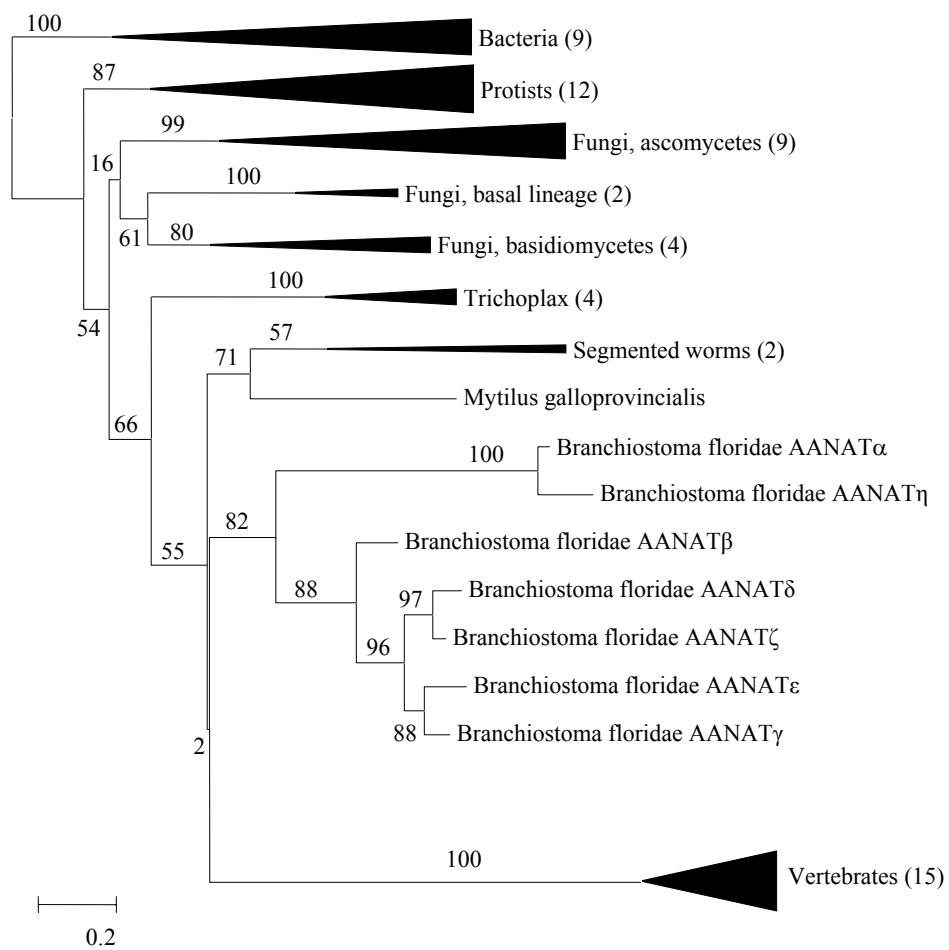

Supplement: Additional file 12 — Topology tree - normal view. Reconstructed constrained large-scale taxonomic topology tree of selected AANAT sequences - normal view based on model shown on Additional file 11. The scale bar represents the number of substitutions per position; numbers in parenthesis show number of species. The number at internal branches shows the bootstrap support (%). [file 1471-2148-10-154-S12.PDF]

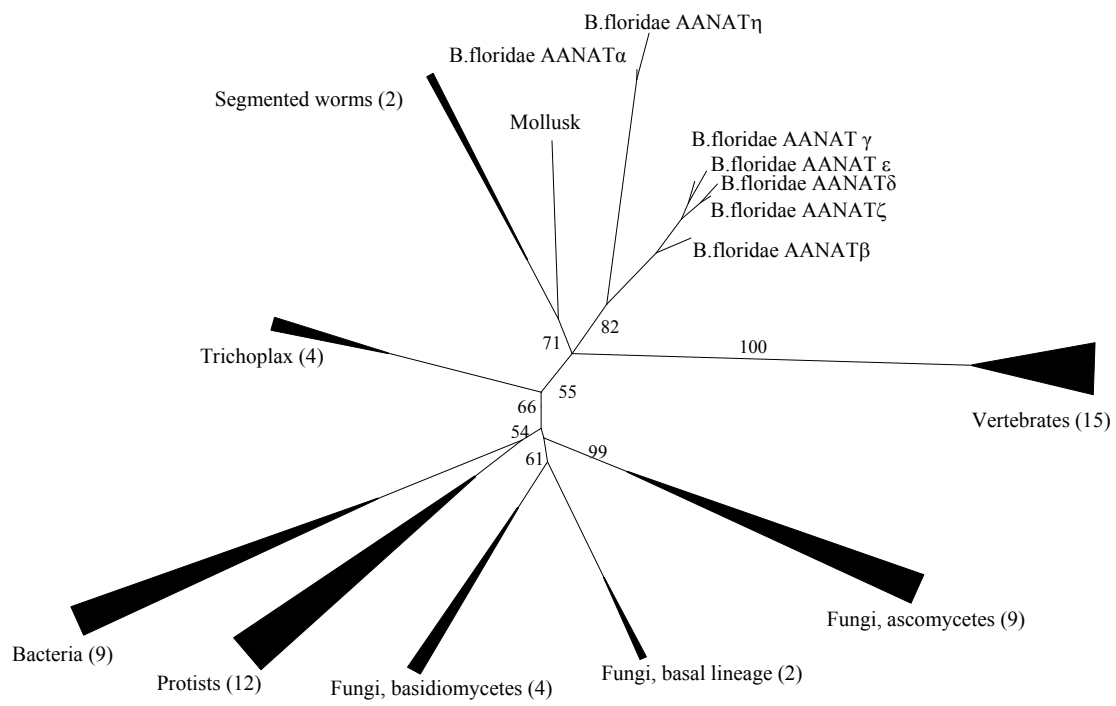

Supplement: Additional file 13 — Topology tree - radiation view. Reconstructed constrained large-scale taxonomic topology tree of selected AANAT sequences - radiation view based on model shown on Additional file 11. The scale bar represents the number of substitutions per position; numbers in parenthesis show number of species. The number at internal branches shows the bootstrap support (%). [file 1471-2148-10-154-S13.PDF]

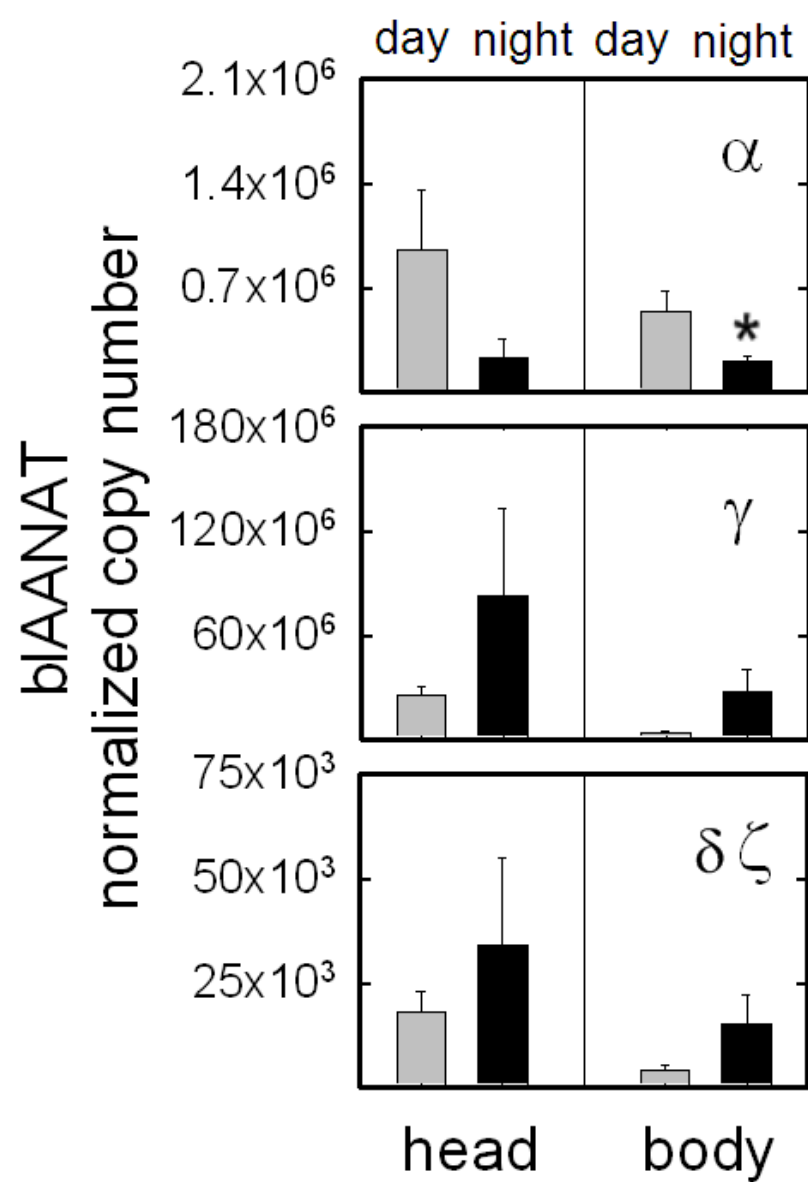

Supplement: Additional file 14 — Comparison of day/night expression of three blAANATs detected by qPCR in the head and body of amphioxus. Primers used are given in Additional file 1. Asterisk indicates statistical significance of day/night differences. Data are presented as the mean ± S.E.M. (N = 3). For further details see Methods. [file 1471-2148-10-154-S14.PDF]

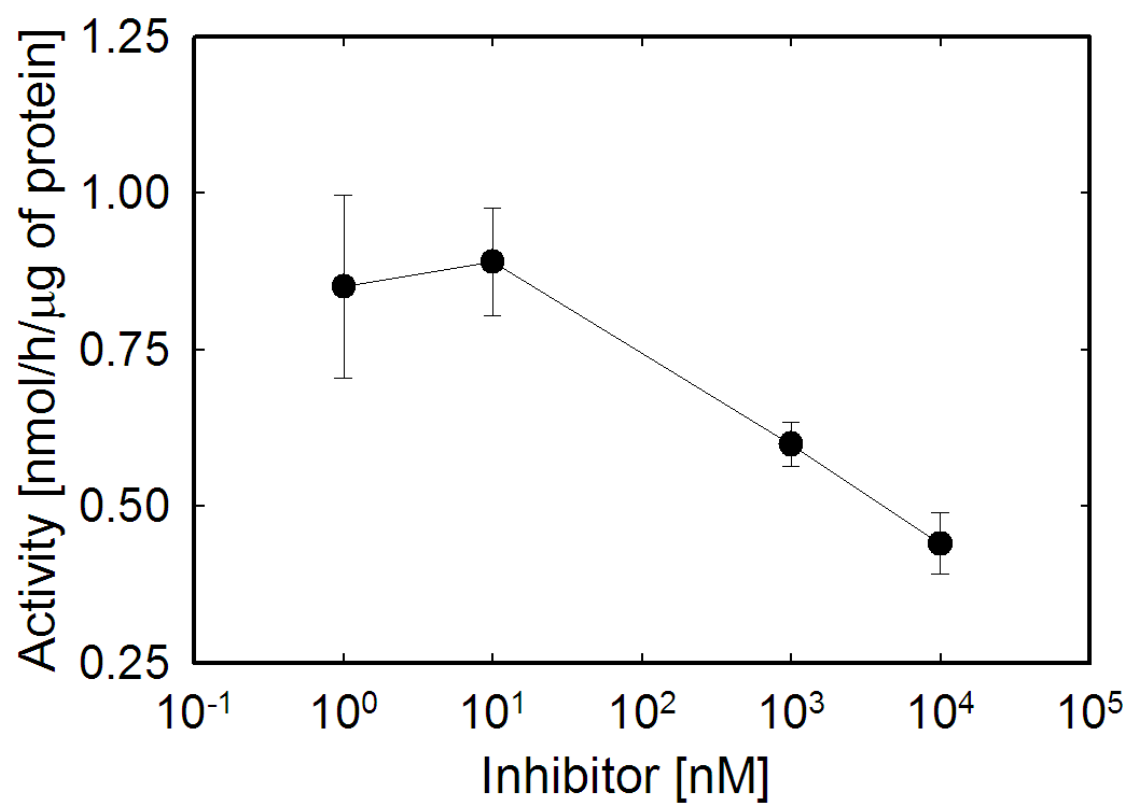

Supplement: Additional file 15 — Inhibition of acetylation activity. Inhibition of acetylation activity in homogenate from amphioxus heads at pH 8.5 using 10 mM PEA and 0.5 mM AcCoA as a substrate and various concentrations of CoA-T as an inhibitor. Data are presented as the mean ± S.E.M. (N = 3). [file 1471-2148-10-154-S15.PDF]
