# Supplementary material for: Evolution of AANAT: expansion of the gene family in the cephalochordate amphioxus
Source: BMC Evol Biol. 2010 May 25;10:154. doi: 10.1186/1471-2148-10-154 (PMC2897805; doi:10.1186/1471-2148-10-154)
Supplement: Additional file 4 — Comparison of deduced amino acid sequences of characterized AANATs to reference genomic sequences. A. AANATα. B. AANATδ'. (g) denotes derived from the published B. floridae genomic sequence. (c) denotes cloned from B. floridae head cDNA, and are the full length recombinant proteins used for enzyme characterization. (f) denotes a partial fragment cloned from B. lanceolatum head cDNA and used as a probe for in toto hybridization. Highlighted residues are those that differ from the published B. floridae genomic sequence. "--", gap inserted to preserve alignment; "...", missing sequence. [file 1471-2148-10-154-S4.PDF]

A

```
bfAANATα(g)  MAEEVFPIHEADSMLSQSSDGLFKQYPRTEEVIRVICNEDELKAAWTLRDKCFPADEYVSLERYQELYHAAPHLCFGYFD 80
bfAANATα(c)  MAEEVFPIHEADSMLSQSSDGLFKQYPRTEEVIRVICNEDELKAAWTLRDKCFPADEYVSLERYQELYRAAPHLCFGYFD
blAANATα(f)                                                   ...FPADESVSLERYQEHYRAAPHLCFGYFD

bfAANATα(g)  GDKVRGFLRGASQKADHFAPDSIGTGVVHDPDGETMVLHLLCVEEQYRRRGIGQGLMKAFIDYVKAKETKVKRIILICHA 160
bfAANATα(c)  GDKVRGFLRGASQKADHFAPDSIGTGVVHDPDGETMVLHLLCVEEQYRRRGIGQGLMKAFIYYVKAKETKVKRIILICHA
blAANATα(f)  GEKLRGFLRGGSQKGEHLPPDSCGA--LHDPDGETMVLHLLCVEEQSRRRGIGQALMKAFIDYVKAKETKVKRVILMCHA

bfAANATα(g)  ELIPVYTRVGFTLVGRAEVKFGKRSWYECCLDLTTYDSVAADDAFQSKYVSMTTDNVPVTTGNANGSTISLESEEEFEEL 240
bfAANATα(c)  ELIPVYTRVGFTLVGRAEVKFGKRSWYECCLDLTTYDSVAADDAFQSKYISLTTDNVPVTTGNANGSTISVESEEEFEEL
blAANATα(f)  ELIPVYTRVGFTLVGRAEVKFGKRSWYECCLDLTT...

bfAANATα(g)  KALDYSD 247
bfAANATα(c)  KALDYSD
blAANATα(f)
```

B

```
bfAANATδ' (g)  MAFMGNSYLTNKALEENIRPLQSAQEVQQAYRLDHASFPKDYEDLPLESWMAVYNEDRRLILGYFKSDKLLGFISASLSD 80
bfAANATδ' (c)  MAFMGNSHLTNKALEENIRPLQSAQEVQQAYRLDDASFPEDYEDLSLETLMAVYNEERRLILGYFKSDNLLGFIGASLSD
blAANATδζ(f)                                           ...PEDYEDLPIETWMTVYNEERSLLLGYFKSEELLGLVGASMSD

bfAANATδ' (g)  VDHYTKEAMNNHVPHGQTICIHSVCVEQNVQHQGIATKLLKEFVHNVKGGSFPEAKRICLICQEYLIPLYMKIGFVLIGL 160
bfAANATδ' (c)  VDHYTKEAMNNHVPHGETICIHSICVEQNAQRQGIATKLLKEFVHNVKGGSFPEAKRICLICHEYLIPLYTKIGFVLIGL
blAANATδζ(f)  ADHYTKEALRTHVSHGETICINSLCVDQNVQRQGIATRLLHKFVLNVKG-SFPKAKRICLI...

bfAANATδ' (g)  SEVVHGKEPWYECILEL 177
bfAANATδ' (c)  SEVVHGKEPWYECILEL
blAANATδζ(f)
```
