# Supplementary material for: Evolution of AANAT: expansion of the gene family in the cephalochordate amphioxus
Source: BMC Evol Biol. 2010 May 25;10:154. doi: 10.1186/1471-2148-10-154 (PMC2897805; doi:10.1186/1471-2148-10-154)
Supplement: Additional file 5 — List of species used for construction of phylogenic tree. List of species containing AANAT homologs used for the construction of the phylogenetic tree shown in Figure 2. "gi" numbers precede the species name, where available; the source of the sequence is given in parentheses. Sequences for which "gi" numbers are not available are given in Additional file 6. [file 1471-2148-10-154-S5.PDF]

BACTERIA:

Firmicutes:
153811594|Ruminococcus_obeum (RefSeq)
15004814|Clostridium_acetobutylicum (RefSeq)
125625097|Lactococcus_lactis (RefSeq)
82752239|Staphylococcus_aureus (RefSeq)
Proteobacteria:
70731909|Pseudomonas_fluorescens (RefSeq)
88799400|Reinekea_sp. (RefSeq)
54303205|Photobacterium_profundum (RefSeq)
Actinobacteria:
23464756|Bifidobacterium_longum (RefSeq)
Bacteroidetes/Chlorobi group:
146301773|Flavobacterium_johnsoniae (RefSeq)

METAZOA:

Placozoa:
196006415|Trichoplax_adhaerens (RefSeq)
196017385|Trichoplax_adhaerens (RefSeq)
196006419|Trichoplax_adhaerens (RefSeq)
196006417|Trichoplax_adhaerens (RefSeq)
Annelida, Polychaeta:
Capitella_sp.I (JGI, genome project)
Alvinella pompejana (EST)
Mollusca:
Mytilus_galloprovincialis (EST)
Cephalochordata:
Branchiostoma_floridae_alpha (JGI, genome project)
Branchiostoma_floridae_eta (JGI, genome project)
Branchiostoma_floridae_beta (JGI, genome project)
Branchiostoma_floridae_delta (JGI, genome project)
Branchiostoma_floridae_zeta (JGI, genome project)
Branchiostoma_floridae_gamma (JGI, genome project)
Branchiostoma_floridae_epsilon (JGI, genome project)
Vertebrata:
170296067|Scyliorhinus_canicula (GenBank)
18858241|Danio_rerio (RefSeq)
41152330|Danio_rerio (RefSeq)
157278427|Oryzias_latipes (RefSeq)
157278457|Oryzias_latipes (RefSeq)
185133507|Oncorhynchus_mykiss (RefSeq)
185133930|Oncorhynchus_mykiss (RefSeq)
31747568|Xenopus_laevis (GenBank)
45384042|Gallus_gallus (RefSeq)
149637697|Ornithorhynchus_anatinus (RefSeq)
126308683|Monodelphis_domestica (RefSeq)
57164193|Ovis_aries (RefSeq)
29135307|Bos_taurus (RefSeq)

4501845|Homo_sapiens (RefSeq)
6752938|Mus_musculus (RefSeq)

PROTISTS:

Bacillariophyta (diatoms):
223999789|Thalassiosira_pseudonana (RefSeq)
219128877|Phaeodactylum_tricornutum (RefSeq)
Chlorophyta (green algae):
116056306|Ostreococcus_tauri (embl)
145356415|Ostreococcus_lucimarinus (RefSeq)
219806964|Chlamydomonas_reinhardtii (GenBank)
226523548|Micromonas sp.RCC299 (GenBank)
Volvox_carteri (JGI, genome project)
Rhodophyta (red algae):
Gracilaria_changii (EST)
Oomycetes:
Phytophthora_ramorum (JGI, genome project)
Phytophthora_sojae (JGI, genome project)
Heterolobosea:
Naegleria_gruberi (JGI, genome project)
Haptophyceae:
Emiliania_huxleyi (JGI, genome project)

FUNGI:

Basal fungal lineages:
Phycomyces_blakesleeanus (JGI, genome project)
Rhizopus_oryzae (database Rhizopus oryzae, genome project)
Basidiomycota:
220723540|Postia_placenta (GenBank)
71003187|Ustilago_maydis_521 (RefSeq)
169856590|Coprinopsis_cinerea (RefSeq)
170116700|Laccaria_bicolor (RefSeq)
Ascomycota:
19114257|Schizosaccharomyces_pombe (RefSeq)
50546791|Yarrowia_lipolytica (RefSeq)
71001602|Aspergillus_fumigatus (RefSeq)
68482097|Candida_albicans (RefSeq)
50425679|Debaryomyces_hansenii (RefSeq)
6320276|Saccharomyces_cerevisiae (RefSeq)
50308813|Kluyveromyces_lactis (RefSeq)
46110206|Gibberella_zeae (RefSeq)
39971637|Magnaporthe_grisea (RefSeq)
