# Supplementary material for: Evolution of AANAT: expansion of the gene family in the cephalochordate amphioxus
Source: BMC Evol Biol. 2010 May 25;10:154. doi: 10.1186/1471-2148-10-154 (PMC2897805; doi:10.1186/1471-2148-10-154)
Supplement: Additional file 6 — FASTA sequences of AANAT homologs. FASTA sequences of those of AANAT homologs used for the construction of the phylogenetic tree shown in Figure 2, but not having "gi" numbers, (as listed in Additional file 5). [file 1471-2148-10-154-S6.PDF]

**Annelida, Polychaeta:**

>Capitella_sp.I
MDFGPATFIKAEQVHEAHALETEAFSPDEAESLETFQRRQNEAPELFIGLYEEGTTNLIA
FICSTRSAGPTWTTQSMKTHLPLGECVCIHSVVVAKQHRKKGIARKLLSFYETHLLTLNP
KPSKIVFVCKENIVDFYKYLGYSYLGPSSEVYGSARWFSMEKLL

>Alvinella_pompejana GO220539
MDSQVAYKIRPLRSDEIKQAHILEEEGYPPDEAASFDTLKYRQKEAPDLFLGLFEDNQLL
AFICGTRYLGPRFRAESMKCHDPDAPCVCIHSVCVKKDKRRQGLALQLLKAYHEHVKETQ
SRVIKMMLLCKARLIPLYTRVRITGMKWSSIYDDLIISKVNCFTNMIYNL

**Mollusca:**

>Mytilus_galloprovincialis FL488956
MYTFDYLDIRPLHKDDINVAFDLEIEGYPEDEAATYDILNYRHTEAPELNRGCFHGDELI
GFVSATRYHEDTLKDEAMNMHIPNGESVCIHSVCVKESRRRQGVATHMLKEFIHYVKHEE
KDAHRILLICKSKLIPLYTRAGFIFKCKSNVVHGKETWYELEVQLKSRENCEELIYNGY

**Cephalochordata:**

>bfAANATα
MAEEVFPIHEADSMLSQSSDGLFKQYPRTEEVIRVICNEDELKAAWTLRDKCFPADEYVS
LERYQELYHAAPHLCFGYFDGDKVRGFLRGASQKADHFAPDSIGTGVVHDPDGETMVLHL
LCVEEQYRRRGIGQGLMKAFIDYVKAKETKVKRIILICHAELIPVYTRVGFTLVGRAEVK
FGKRSWYECCLDLTTYDSVAADDAFQSKYVSMTTDNVPVTTGNANGSTISLESEEEFEEL
KALDYSD

>bfAANATβ
MAEGNVRPLQCGEEVEQASILESAGYPADEAASLETLQARHTAESRLFIGYFENEKLLGF
VCATSTDADRLTEESMHTHIPHGETICIHSVCVDQSVQRQGIATKLLKEFVHNVKGSFPD
AKRICLICHEYLIPLYTKAGFVLVGLSEVVHGKEPWYDCVMEL

>bfAANATγ
MAQENIRLLQCVQEVQQASSLEYACFPADEAEPLETWMRRYSEERRLILGYFKGDKLIGF
IGATMSDVDHYTKEAMNTHVPHGETICIHSVCVDQSVQRQGVATTLLKEFVQHVKGRFTE
AKRICLISHEYLLPLYTKIGFVLIGLSEVVHGKEPWYECILEL

>bfAANATδ
MAFMQNSHSTNKVEEEIRPLRCVQEVRQAYILDDASFPEDYEDLSLEALMAVYNEEKRLI
LGYFKNDKLLGFIGASLSDVDHYTKEAMNTHVPHGQTICIHSICVEQNVQRQGIATKLLK
EFVHNVKGGSFPEAKRICLICQEYLVPLYTKEGFVLIGLSEVVHSKEPWYECILEL

>bfAANATε
MTEEHIRPLQCLQEVHQDSRMEYACFPADEAEPLETWMRRYSEEKRLILGYFKSNKLLGF
IGATISDVDHYTKETMNTHVPHGQTICIHSISVDQNVQHQGIATKLLKEFVQHVEGCFPE
VKRICLICHEYLIPLYTKIRFVLIGLSEVVHGKDPWYECILKL

>bfAANATζ
MASMNSHSANKAEGEIRPLRCVQEVQQAYKLDDASFPEDYEDLPLETLMAVYNEERRLIL

GYFKSDKLLGFIGATKSDVDHYTKEAMNNHVPHGQTICIHSICVDQSVQRQGIATKLLKE
FVHNVKEGTFPEAKRICLICQEYLIPLYTKAGFVLIGLSEVVHGNEPWYECILEL

>bfAANATη
MTGGCPEIVHMTEAASLIHESGSMQDMLSSSSDGLFNQNPRSEEVIRVIRNENELKAAWT
LRDKCFRFNRFVSLERYQELYRAAPHLCFGYFDGEKLRGFLRGASQKADHFTPDFTGTGN
VHDPDGETMVLQLLCVEEHYRRRGIGTRLIKAFIDHVKAKETKVKRIIVKCYAELIPVYT
RVGFTLVGRVEIKAGSRYECCLDLEGSRTQYLCATPGSMTWVHKIVRINLLTKR

**Chlorophyta (green algae):**

>Volvox_carteri
MATSEEESNHILEQYQSLGNTIFFAPVQPEHLIRIHELESSSYPADEAATFQKLEFRILS
APNVFMVAMQCRDGGAEPEVVGYVCGTCTNAGRLTHESMATHDQEGALLCIHSVVVEAGL
RRKGLATRLLRAYVPYVQATTPHLQAIRLICKQDLIGLYEKAGFTLAGPSDVVHGRDPWF
EMVLELPSGDS

**Rhodophyta (red algae):**

>Gracilaria_changii DV965953
METMPTHAVARVFELEKDSYPADEAASLETIEYRASVTEGLCQIAEDASTKDIIGFVTAT
AAPKGTERMSASMMKSHSANGKVVCIHSVVVDGRFRRRGVGRQMLDAYVNNIKKTGQYER
MLLISKPYLTHFYEACGFTVIGESAISHGKDCWIEMGMTLRFP

**Oomycetes:**

>Phytophthora_sojae
MPSPSHLRLALLTEERDIQRVAALEAASYPADEAASESGIRFRQQNAGAFFWAAYLPTNG
DSETLVGFVNGTLTARHELDDESMSQHDPHGSLLCIHSVVVDPAFRRRGLAAQMLKRYVR
LVCDLQPQVTRIMMIAKAYLVKFYVGCGFSVTRLSPVVHGQDPWFELELDCQAARCPPMI
QVRDKDHCGKVLLLLTCDACFRWMHSRARRFRATRRLWCCCRRLRSTSPRRRSGCSEWLL
RTT*

>Phytophthora_ramorum jgi|Phyra1_1|81034|
MKTSNLLRLAQLTQEDDILRVAALEAASYPADEAASDSGIRFRQKSAGAFFWAAYLPTDKEQEILVGFVN
GTLTARQELDDESMSQHDPHGSLLCIHSVVVDPAFRRRGLAAQMLKRYVRIMCEQQPQVKRIMMIAKAYL
VKFYVDCGFAVTRLSPVVHGQDPWLELELNCETARLPPVIQVDAFSSETFQGNPAAVVLLTPTVYHKPEA
SEWMQRVALENNLSETAYTALRERTTTTPDEVVEYDLRWFTPAAEVKLCGHATLSTAFTLHDAGLVATSQ
TIHFHTLSGVLVCRFEVEADTQKLLVLMDFPEQPAESAGPSVVLSEVAKALGISSDAIVDVKRATTDLLV
RVTPEAFAALQPDFVELAKTDVRGFVVTAEMKDNALGVDIQSRFFGPRVGVNEDPVTGSAHCALGPYWAP
LLKKTTIKAQQFTPVRGGFITLDLVAAGPGRVLLKGEGVIVLRVNPFGELTLTDEDRAQLVKIADALVLA
KFEEYEEHLNNEKRVDLKRWKKITSSGTTTQYLERKNSNPDSKLPALLMTGPLPGTLDENMFGLVSPTIE
AMRIKSSYLDDFSAAAVLATVKEPTLEEPFRSVVVKWMEIDIPGALIGLVRNRDYVYVESSGILHLKNGE
RVGYHLFHSVDFKQAHELPSRVRGNMSFVGIFHQEGPDRTDCRGTGIMDPGGDLIRVMAIMGMVQATMAG
LKYSYCGQMKKLAWLLEQRHAQTKERGAPVTEPVCVTCCKQTKTSKFGKFGKSSSKCKLCFGALCGTCKI
AKKLSFITPDLGLAQRKVIFCVKCLVEATSMDTLEAARQQFVYKKPVQPSVYGSSVASDTVFVTSLAMLI
EITDALIMAKFEEYEEHLNIGKKVDLKRWKKFAKSGPTTSYLERKASSPNSKLPQLLMVGPLPGSLDENM
FGIVNPTIEAMRIKSSYLSDFNAAAVLATVVEPTVDEPFRSVVVKWMEIDIPLASIGLVRNRDYVYVEST
GILHLKNGERVGYHLFHSVDFHQAHELPSRVRGNMSFCGIFHQEAPDRTDCRGTGIMDPGGDMIRAMAVM
GMVQATMAGLKYSYCGQMKKLAWLLEQRQVEAREKGTPAFKPFCVTCMKGVKQSKISGPVHTCKLCFGAV
CNSCKISKKLSFIAPDLTLAQRKVTFCVKCMIDATRMDTQEAAREQFVYKKSVTPAMYGISVVSDMSTCS

ESTMTTRTGYSGSTN*

**Heterolobosea:**

>Naegleria_gruberi
MSSSSTSYLLKPVNVTEHLEIINKYESESYPEDEAATYEKLKYRLENANSVFRGYFTSST
CSDDEEKLIGFVCGTKTQNKILTHDTMSVHDPSPNARTLCIHSVVIQQAYRKKGLGLAML
KSYLQYLKDENLCDKILLLSKSHLIHFYESAGFILNGESNVVHGQEKWYELEYKMN*

**Haptophyceae:**

>Emiliania_huxleyi
MLPALVAPLPLLFRPVSLSLLDAAHAIEAASYPADEAASRDNLEKRLRDAAPYFYGAFDD
SSGALRGFVCGTCTSSEALTEEAMSTHEPDGAVLCIHSVVVDEAYRRRGVGAWMLRSYLA
EVAAAGRVRTVLLLCKPHLEAYYAAAGFASLGDSGVSHGATPWTLMRLGLRTS*

**Basal fungal lineages:**

>Phycomyces_blakesleeanus
MSFLESLKFEPACLLDLDVIDHLEQTSYHPDEAATREKLKNRIEYAAHSGPELFLVAKDKETIVGFVCTT
LSRSDLLTEESMDVHDPEGKTVCLHSVCVAPEYRKRGVATHLLNTWIGILRHHNELQTPKKYERVAILSR
PNLLSLYGSVGFKNLGKSEVVHGPEPWYDCILEL*

>Rhizopus_oryzae
MKFIYTPATPADLDIVSEYEEKSYHPDEAASKEQLKARIGYASQSGPELFTVARDAQDNS
VVGFLCSTLTTNELVTDESMSVHDPNGKTICLHSVCVAPHMRNRGIATELLINWIQQLKQ
INESTKNKKYERVAIMSRPSLMAFYEKVGFKNKGISQVVHGPEPWIDCVLEL*
