# Supplementary material for: Evolution of AANAT: expansion of the gene family in the cephalochordate amphioxus
Source: BMC Evol Biol. 2010 May 25;10:154. doi: 10.1186/1471-2148-10-154 (PMC2897805; doi:10.1186/1471-2148-10-154)
Supplement: Additional file 8 — Average evolutionary distance (substitution per position) between major taxonomic groups for AANAT proteins based on the truncated alignment shown in Additional file7(JTT substitution model, gamma distribution 1). [file 1471-2148-10-154-S8.PDF]

	amphioxus	vertebrates	mollusk	worms	trichoplax	protists	fungi	bacteria
**amphioxus**	-							
**vertebrates**	1.99	-						
**mollusk**	1.17	1.84	-					
**worms**	1.57	1.92	1.84	-				
**trichoplax**	1.64	1.93	1.87	1.71	-			
**protists**	1.70	2.15	2.01	1.64	1.58	-		
**fungi**	1.75	1.95	1.86	1.67	1.68	1.68	-	
**bacteria**	2.12	2.34	2.28	2.14	1.94	1.93	1.93	-
