# Supplementary material for: Evolution of AANAT: expansion of the gene family in the cephalochordate amphioxus
Source: BMC Evol Biol. 2010 May 25;10:154. doi: 10.1186/1471-2148-10-154 (PMC2897805; doi:10.1186/1471-2148-10-154)
Supplement: Additional file 9 — Average differences (p-distance) between major taxonomic groups for AANAT proteins, based on the truncated alignment shown in Additional file7, calculated using the MEGA program[34]. [file 1471-2148-10-154-S9.PDF]

	amphioxus	vertebrates	mollusk	worms	trichoplax	protists	fungi	bacteria
**amphioxus**	-							
**vertebrates**	0.75	-						
**mollusk**	0.59	0.73	-					
**worms**	0.69	0.73	0.64	-				
**trichoplax**	0.70	0.75	0.68	0.69	-			
**protists**	0.71	0.77	0.65	0.70	0.68	-		
**fungi**	0.71	0.74	0.68	0.70	0.71	0.70	-	
**bacteria**	0.75	0.77	0.73	0.76	0.74	0.74	0.74	-
