# Supplementary material for: Evolution of AANAT: expansion of the gene family in the cephalochordate amphioxus
Source: BMC Evol Biol. 2010 May 25;10:154. doi: 10.1186/1471-2148-10-154 (PMC2897805; doi:10.1186/1471-2148-10-154)
Supplement: Additional file 10 — Average percent identity between major taxonomic groups for AANAT proteins based on the truncated alignment shown in Additional file7, calculated from Additional file9. [file 1471-2148-10-154-S10.PDF]

	amphioxus	vertebrates	mollusk	worms	trichoplax	protists	fungi	bacteria
**amphioxus**	-							
**vertebrates**	24.6	-						
**mollusk**	41.3	26.9	-					
**worms**	31.2	27.4	35.9	-				
**trichoplax**	29.6	24.9	31.9	30.7	-			
**protists**	29.3	22.7	35.3	30.3	31.6	-		
**fungi**	29.1	26.5	31.8	29.9	29.0	29.7	-	
**bacteria**	24.9	22.8	26.9	24.1	26.0	26.1	26.2	-
